# Identification of fluorescently-barcoded nanoparticles using machine learning†

**DOI:** 10.1039/d2na00648k

**Published:** 2023-03-23

**Authors:** Ana Ortiz-Perez, Cristina Izquierdo-Lozano, Rens Meijers, Francesca Grisoni, Lorenzo Albertazzi

**Affiliations:** a Institute for Complex Molecular Systems (ICMS), Eindhoven University of Technology PO Box 513 5600 MB Eindhoven The Netherlands l.albertazzi@tue.nl

## Abstract

Barcoding of nano- and micro-particles allows distinguishing multiple targets at the same time within a complex mixture and is emerging as a powerful tool to increase the throughput of many assays. Fluorescent barcoding is one of the most used strategies, where microparticles are labeled with dyes and classified based on fluorescence color, intensity, or other features. Microparticles are ideal targets due to their relative ease of detection, manufacturing, and higher homogeneity. Barcoding is considerably more challenging in the case of nanoparticles (NPs), where their small size results in a lower signal and greater heterogeneity. This is a significant limitation since many bioassays require the use of nano-sized carriers. In this study, we introduce a machine-learning-assisted workflow to write, read, and classify barcoded PLGA–PEG NPs at a single-particle level. This procedure is based on the encapsulation of fluorescent markers without modifying their physicochemical properties (writing), the optimization of their confocal imaging (reading), and the implementation of a machine learning-based barcode reader (classification). We found nanoparticle heterogeneity as one of the main factors that challenges barcode separation, and that information extracted from the dyes' nanoscale confinement effects (such as Förster Resonance Energy Transfer, FRET) can aid barcode identification. Moreover, we provide a guide to reaching the optimal trade-off between the number of simultaneous barcodes and classification accuracy supporting the use of this workflow for a variety of bioassays.

## Abstract 2

We introduce a machine-learning-assisted workflow to write, read, and classify dye-loaded PLGA–PEG nanoparticles at a single-particle level.

## Graphical abstract

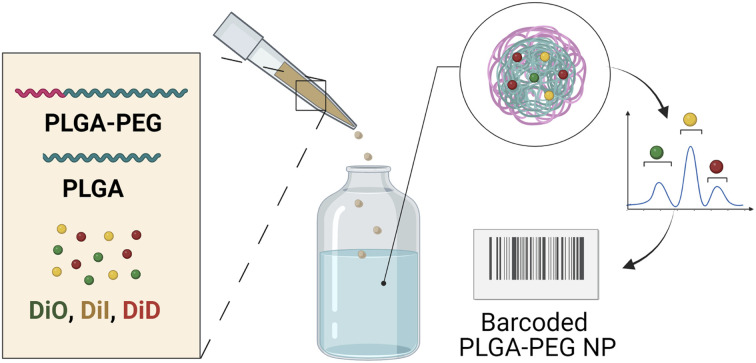

## Introduction

Particle-based biochemical assays are cornerstones of molecular diagnostics, materials, and drug discovery, as well as *in vivo* and *in vitro* molecule and cell tracking.^1^ One of the current focuses in this field is to make these assays high-throughput to increase their speed and the number of tests you can perform per unit of time. An approach to achieving high-throughput assays is to label the particles of interest with readable tags (barcodes), to distinguish them within a mixture, and run multiple assays simultaneously in ‘one-pot’.^2,3^ In the past decades, several strategies have been proposed for information encoding, *e.g.*, morphological,^4,5^ magnetic,^6^ DNA,^7^ optical,^8^ or hybrid/multi-modal^9–12^ barcoding systems. Among these, optical-based methods are widely used, since they offer a high number of codes, multiple encoding parameters, and fast and robust decoding methodologies that can be easily implemented in most applications.^3^ Fluorescent-based barcoding is the most widely used optical modality, with the preferred encoding element being the fluorescent spectra and intensity. Depending on the chosen optical probe and read-out, other features can also be exploited, such as lifetime,^13^ phase-angle,^14^ or probe kinetics.^15^ Unlike intrinsic properties, the intensity varies with probe concentration, with different intensity levels achievable by varying the amount of fluorophore per particle.

To date, fluorescent barcoding has been mostly applied to microparticles^16,17^ due to their high encoding capacity, homogeneity, and ease of fabrication and labeling.^18,19^ The high encoding capacity is mainly attributed to their large volume or surface area available to host a high number of optical probes at a wide range of concentrations, which translates into several narrow and separable intensity levels. Moreover, detection and fluorescence quantification in microparticles can be easily achieved due to the high signal-to-noise ratio (SNR) and large size.

However, in applications where particles are used as labels to study cells and biological processes (*e.g.*, cell labeling, single-virus intracellular trafficking, or receptor tracking^20–23^), nanoparticles (NPs) are preferred or even required. Downscaling objects from micro to nano is not trivial, and barcoding NPs has been proven to be remarkably more challenging. A smaller volume results in less available space to incorporate probes when compared to their micron-size counterpart. This is translated in lower and narrower ranges of intensities, low SNR, and the aggravation of energy transfer processes due to the proximity of dyes within the matrix (*e.g.*, aggregation-caused quenching (ACQ) and Förster Resonance Energy Transfer (FRET), among others). Moreover, nanoparticles are intrinsically more heterogeneous, since small variations in size can lead to big changes in properties. Finally, dye incorporation often influences nanoparticle surface properties, especially when dyes are conjugated to the surface. Since NP reactivity, interaction with the environment, and biological performance are highly influenced by surface properties, preserving the NP surface is pivotal in many applications, such as drug delivery. Some strategies have been successfully proposed to improve the ACQ of encapsulated dyes (*e.g.*, modifying the dye with a counterion prior to encapsulation^24^), but less attention has been given to extracting information from the energy transfer processes in dye-loaded particles^25^ or even using machine learning to distinguish different classes or populations.^9,26^

In this work, we want to explore the challenges in the engineering, imaging, and identification of individual fluorescent nano-barcodes, which preserve their properties after dye incorporation. As a model carrier, we choose Poly Lactic-*co*-Glycol Acid–Poly Ethylene Glycol (PLGA–PEG) NPs, a widely used biocompatible material in countless biomedical applications because of its low toxicity, biocompatibility, and biodegradability.^27^ We encapsulated all possible combinations of three conventional spectrally separated dyes at one of three intensity levels (zero, low, high); leading to a total of 26 barcodes. Within this framework, we explored the feasibility and challenges of barcoding per intensity level and color combination. We found out that nanoparticle heterogeneity is the main limiting factor, even for monodisperse particles with <0.1 polydispersity index (PDI) by dynamic light scattering (DLS). We also observed that barcoding by color combinations gives rise to more distinct fingerprints than barcoding by intensities of the same color. We also hypothesized that information related to energy transfer and spectral overlap can increase the accuracy of the barcode identification. For this, we implemented a multichannel-detection strategy, which collected not only information about direct excitation and emission, but also about cross-excitation and cross-emission. To enable the identification of barcodes, we trained a machine learning classifier, or barcode reader, which was capable of classifying individual nanoparticles. We then investigated how the accuracy of the classifier increased by systematically removing the less precise classes. With this information, we studied the tradeoff between the number of barcodes and the classification accuracy, thereby providing a guide to choose the proper barcoding strategy depending on the application. We believe that this approach and the fundamental understanding provided in this work could aid in the design of barcode schemes that can be easily integrated into a variety of fluorescent bioassays to increase their throughput.

## Results and discussion

### Barcoding strategy scheme

**Fig. 1:**
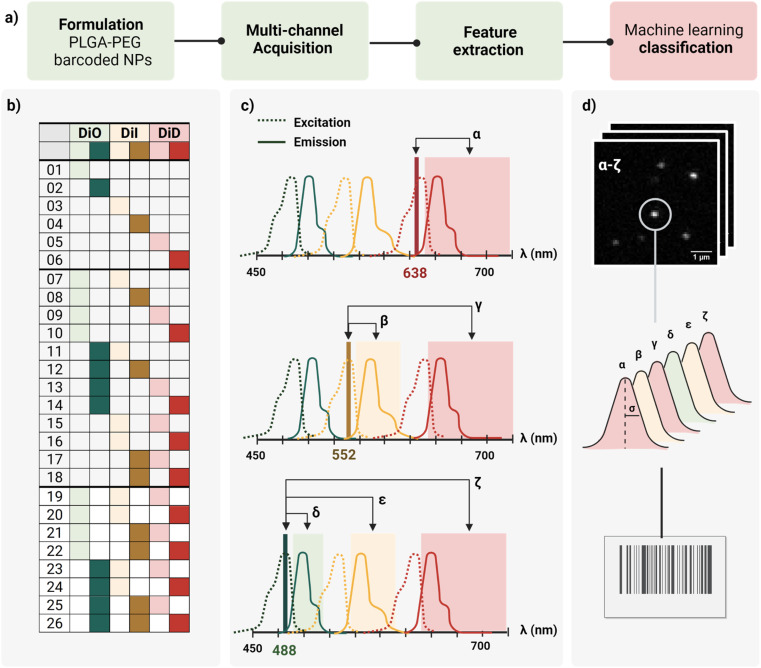
(a) General workflow from Poly Lactic-*co*-Glycol Acid (PLGA) Poly Ethylene Glycol (PEG) nanoparticles (NPs) formulation to machine learning classification. (b) Dye encapsulation scheme of the 26 barcodes proposed using three moderately separated optical dyes (DiO, DiI, DiD) at three concentration levels (0, 1 μM, 10 μM or zero, low, high) to form single, dual, or triple mixtures. (c) Schematic representation of the six detection channels based on spectral properties of the dyes (α–ζ): including three main channels – α (DiD, 638 ex., 648–750 em.), β (DiI, 552 ex. 563–620 em.) and δ (DiO, 488 ex., 498–538 em.) – and the three additional channels (γ, ε, ζ) to capture spectral overlap and dye interactions. (d) Illustration of feature extraction (intensity and sigma of the fitted Gaussian) from confocal raw images (α–ζ), and classification of the nano-barcodes based on those features.

In this work, we report a workflow to write, read, and classify fluorescently barcoded nanoparticles at a single-particle level, as schematically reported in Fig. 1a. PLGA–PEG NPs were formulated by bulk nanoprecipitation, encapsulating different mixtures of three spectrally-separated organic dyes (DiO, DiI, DiD) in three specific concentrations (0, 1 μM, 10 μM or zero, low, high) yielding 26 possible combinations or N classes (Fig. 1b), according to *N* = *n*^m^ − 1, where *n* is the number of intensity levels and *m* the number of dyes. For each barcode (class), more than 2.000 single nanoparticles were imaged with confocal microscopy in six channels (designated with Greek letters α–ζ), which were defined based on the spectral properties of the dyes (Fig. 1c), capturing not only the characteristic signal of individual dyes (direct excitation and emission) but also the spectral overlap and interactions between dyes (*e.g.*, cross-excitation, cross-emission, FRET, and other non-radiative interactions), given our optical set-up. The resulting confocal images were analyzed to extract the integrated intensity and sigma of the fitted Gaussian for each nanoparticle. With this reading procedure, we obtained 14 features for every individual nanoparticle that were then analyzed using Principal Component Analysis (PCA) and a machine learning classification algorithm (Fig. 1d) to assign every detected particle to the correct class.

### Particle formulation and ensemble characterization

The first challenge to successfully engineer barcoded nanoparticles is the incorporation of the probe into the carrier in a way that yields sufficient signal to be detected without perturbing key properties, such as size and surface chemistry. To preserve the carrier surface, we chose dye encapsulation over surface conjugation. This method is also more time- and cost-effective, since probe incorporation is achieved *in situ* in a single step, during the formulation process of the NPs by bulk nanoprecipitation.

However, not every dye will incorporate with the same efficiency. PLGA–PEG is a *block* co-polymer that self-assembles into nanospheres with a hydrophobic core (PLGA) and a hydrophilic surface (PEG). Previous work in our group studied the relation between Encapsulation Efficiency (EE) and physicochemical properties of three conventional red dyes (DiD, NileRed, Doxorubicin), and found that EE was increased with the degree of hydrophobicity of the dye.^28^ For this work, we chose the dye with the highest EE (DiD), and we added to the color panel two more dyes of the same family with similar chemical structures and properties (DiO, DiI). Apart from EE, it is important that the dye keeps its fluorescence once incorporated into the core since fluorescence is highly influenced by the matrix. To assess dye encapsulation, we formulated PLGA–PEG NPs with a range of dye concentrations from 0.1 μM to 100 μM and measured fluorescence intensity with bulk spectroscopy (Fig. S1†), and single-particle intensity with Total Internal Reflection Fluorescence (TIRF) microscopy (Fig. S2†). These results confirmed dye encapsulation and particle detection at a single-particle level. Ensemble results (bulk spectroscopy, Fig. S1b†) showed an increase in fluorescence with an increase in dye concentration until saturation around 50 μM. After this value, increasing the concentration of added fluorophore leads to a marked decrease in intensity, which likely occurs due to energy transfer processes such as Aggregation-Caused Quenching (ACQ). Finally, we checked whether dye encapsulation influenced nanoparticle properties by DLS and zeta potential (Fig. S3†). We confirmed that carrier properties did not vary significantly with the addition of dye. Based on the previous results, the dyes DiO, DiI and DiD were chosen to be encapsulated in three intensity levels 0, 1 and 10 μM inside PLGA–PEG NPs, yielding the 26 possible combinations depicted in Fig. 1b. Bulk physicochemical characterization of these barcodes is available in the ESI† (Table S1, Fig. S4 and S5†).

### Single-particle optical characterization

The second challenge is the detection and quantification of the barcoded nanoparticles. To optimally image individual nano-barcodes on a glass substrate, they must be immobilized on the surface to avoid fluctuations in the fluorescence signal due to Brownian motions. Second, they should be distributed at a medium-to-low density of particles per Field Of View (FOV) to ensure that the detected signal originates only from one particle. Finally, a sufficient SNR is required for proper identification: techniques such as Confocal microscopy or TIRF possess enough sensitivity to achieve proper SNR values for detection and later quantification.

As every object is below the diffraction limit of light, nanoparticles appear as bright spots, with a corresponding point spread function (PSF), whose size is influenced by the wavelength of the excitation light, the numerical aperture of the objective and the actual size of the object.^29^ Based on theoretical calculations, we estimated the Full Width at Half Maximum (FWHM) of our nanoparticles to be around 200 nm, about twice as big than the particles' “real size”. We used this value as a reference for proper identification of single nanoparticles. Confocal microscopy was chosen as the imaging modality for the barcoded nanoparticles due to the capability to resolve single nanoparticles as a PSF with a good SNR (Fig. S6†), and the possibility to implement a multi-channel detection strategy, by defining specific laser-detection window combinations (channels).

As schematically depicted in Fig. 1c, we defined six acquisition channels, each of them designated with a Greek letter (α–ζ). Three of the channels (α, β, δ) collect the direct fluorescence signal of each of the three fluorophores at their optimal excitation wavelength: α for DiD, β for DiI, and δ for DiO. The other three additional channels (γ, ε, ζ) aim to capture a combination of spectral overlap and possible dye interactions: γ for DiI cross-emission or bleed-through, DiD cross-excitation, DiI–DiD energy transfer; ε for DiO cross-emission or bleed-through, DiI cross-excitation, DiO–DiD energy transfer; and ζ for DiI-cross emission due to energy transfer from DiO or DiI cross-excitation. Depending on the dye combination, we expect to detect different patterns across the six channels. For instance, when having one of the three dyes present, we expect to collect most of the signal from the corresponding main channel, and a variable fraction of this signal from the additional channels. This way, we aim to capture the optical fingerprint of the barcoded nanoparticles and to investigate whether information related to energy transfer and optical overlap is useful to separate the barcode populations, and to which extent.

**Fig. 2:**
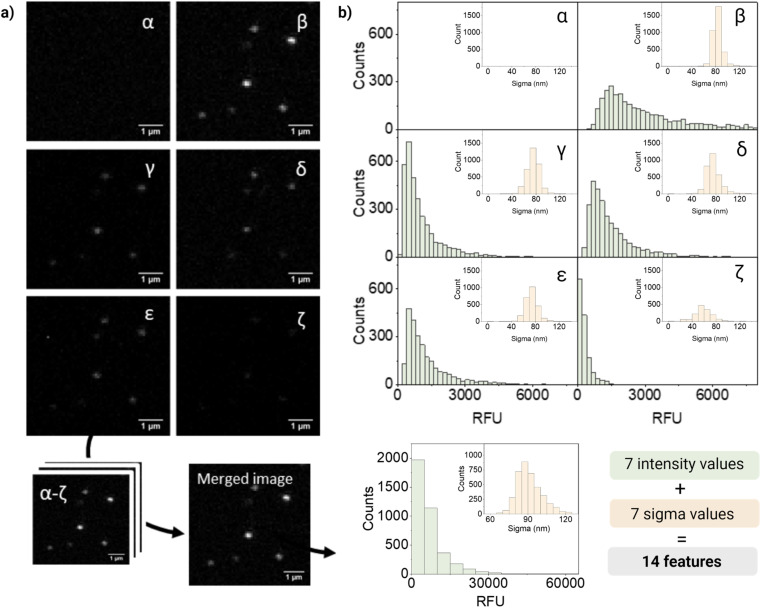
(a) Representative confocal images of barcode 12 (10 μM DiO, 10 μM DiI) in channels α–ζ and merged channel (400% zoomed in): barcoded nanoparticles appear as point spread functions that can be fitted to a Gaussian function and which intensity and sigma can be quantified. (b) Distribution of intensities and sigma values of the nanoparticle population for each of the six acquisition channels (α–ζ) and the merged one. The resulting 7 values of intensity and sigma per particle constitute the 14 features that can be used in the machine learning algorithm to discriminate barcodes/classes.

For quantification and feature extraction from the acquired images (Fig. 1d), we used ThunderSTORM,^30^ an Image-J plug-in generally used for single molecule analysis. This software identifies the centroid of each PSF, fits it to a Gaussian function and extracts the integrated intensity and sigma (width of the function at half height) for each detected spot. As not every particle will appear in every channel, all channels are merged into a reference channel (Fig. 2a), which is used to find the coordinates of every particle in the FOV and assign them an ID. The particle coordinates are then used to match the corresponding values of intensity and sigma of the same particle in each of the acquisition channels. Finally, the resulting dataset includes: NP ID, its coordinates, barcode (class) and the corresponding 14 features (7 intensity values and 7 sigma values extracted from the six acquisition channels and the merged channel) (Fig. 2b).

Fig. 2 shows representative confocal images and the corresponding quantification of barcode 12, a dual mixture of DiO and DiD at high intensity. As expected, most of the fluorescence intensity was collected from two of the main detection channels corresponding to DiO and DiI (β and δ, respectively), a fraction of signal from the additional channels (γ, ε, ζ), and no intensity from the DiD channel (α). The intensity measured in channel γ could be mainly attributed to DiI cross-emission or bleed-through, whilst in channel ζ cross-emission and cross-excitation of DiI are likely to be the most contributing factors. Ultimately, in channel ε, that combines the excitation laser of DiO with the emission window of DiI, a fraction of the measured intensity could be attributed to DiO bleed-through and DiI cross-excitation and to the energy-transfer between these two fluorophores. FRET is possible when dyes are in close proximity (within 10 nanometers from each other) and their emission and excitation spectra overlap, which would be the case of the encapsulated dyes, being DiO the donor and DiI the acceptor.

Interestingly, when looking at single-particle intensity distribution, we noticed a significant overlap between two different intensity levels of the same fluorophore, whilst in bulk these intensity levels seem to be very well separated (Fig. S7†). This heterogeneity can also be seen in the distribution of intensities in Fig. 2b. Heterogeneity in size and features becomes critical when evaluating single nanoparticles and only becomes evident when analyzing particles at a single-particle level. We believe that the variations in intensity between nanoparticles with the same signature (barcode/class) could be attributed to a variety of factors. Firstly, even though our nanoparticles are considered highly monodispersed (PDI < 0.1), small fluctuations in size can have a considerable influence on the effective encapsulation of the dye (since the dye concentration varies with *r*^3^ of the particle). In addition, dye encapsulation is a stochastic process and, during this process, the dyes can be oriented in diverse ways, some of which can favor their aggregation through π–π stacking causing quenching of the probe. Finally, interaction between dyes leads to phenomena like FRET, influencing the fluorescent signal.

The non-linearity of dye–dye and dye–matrix interactions, as well as the heterogeneity between particles, challenges the classification of nano-barcodes. Among these “undesired” dye interactions, multicolor FRET is generally seen as the main impediment to discriminate dye-loaded particles, since no mechanistic model can describe the phenomenon in its complexity.^25^ One could also try to minimize or virtually eliminate some of these effects by tuning the optical setup (*e.g.* excitation source, emission filters) or by establishing intensity thresholds to filter the signal coming from these interactions, which in turn would result in loss of signal and information. However, we hypothesized that the information related to these phenomena can be exploited to our advantage to encode and decode the barcodes using machine learning. This type of methodology allows us to find patterns within data that are not intuitive or clear for humans.

### Optical fingerprint visualization

**Fig. 3:**
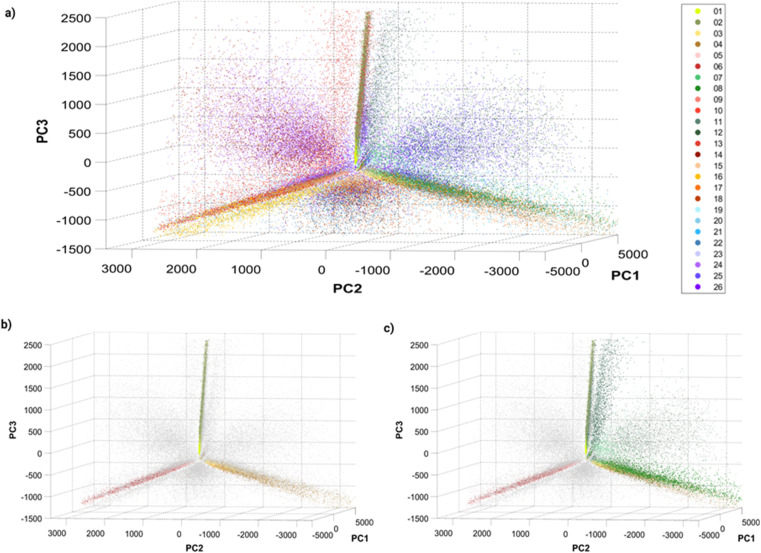
Principle Component Analysis (PCA) of single barcoded nanoparticles: three-dimensional plots of principal components 1, 2 and 3 with (a) all 26 barcodes colored, (b) single colors colored, and (c) single and dual colors (Dil–DiO mixtures) colored. The variances explained by the different principal components (PC) are: PC1 = 80.4087%, PC2 = 13.0798% and PC3 = 5.6811%. Different perspectives on the PCA plot can be found in Fig. S8† and online.

Before training a machine learning algorithm for classification, the 14 features previously acquired per particle were turned into a 14 dimensional dataset. The 14-D data was visualized in a 3D space using Principal Component Analysis (PCA, Fig. 3), in which a single dot corresponds to an individual barcoded nanoparticle.

The visualization of the complete set of 26 barcodes (Fig. 3a) highlights the discussed heterogeneity and overlapping between classes. Despite this heterogeneity, the classes seem to have a preferential orientation in this space. Fig. 3b displays the six single-color barcodes (DiO, DiI, DiD) at low or high intensities (1, 10 μM). The three colors seem to align forming an axis towards a common vertex. The low intensity barcodes seem to be more localized in space, closer to this central vertex, where they overlap with the high intensity population that also displays a higher spread. This increased heterogeneity in intensity with increasing dye concentration can also be observed in the intensity histograms (Fig. S7†). Interestingly, dual mixtures of DiO and DiI appear in a plane between their two corresponding single colors (Fig. 3c). Moreover, the higher the amount of one color in the mixture, the closer the barcode gets to that single color. For instance, barcode 8 (1 μM DiO, 10 μM DiI) is closer to barcodes 3–4 (DiI-only), while barcode 11 (10 μM DiO, 1 μM DiI) is closer to barcodes 1–2 (DiO-only). The same trend can be observed with DiI–DiD and DiO–DiD mixtures. This rational organization in space may be used for anticipating the position of new barcodes and guiding the design of new combinations. Despite this seemingly predictable orientation of barcode populations in space, there is a substantial overlap between barcodes that challenges their identification. As discussed earlier, the intrinsic heterogeneity of nanoparticles in size and dye encapsulation together with energy transfer phenomena hinder barcode identification. Therefore, the establishment of an automated machine learning classification method to identify the barcodes becomes crucial for data that cannot be easily separated visually.

### Machine learning classification and barcode reader

**Fig. 4:**
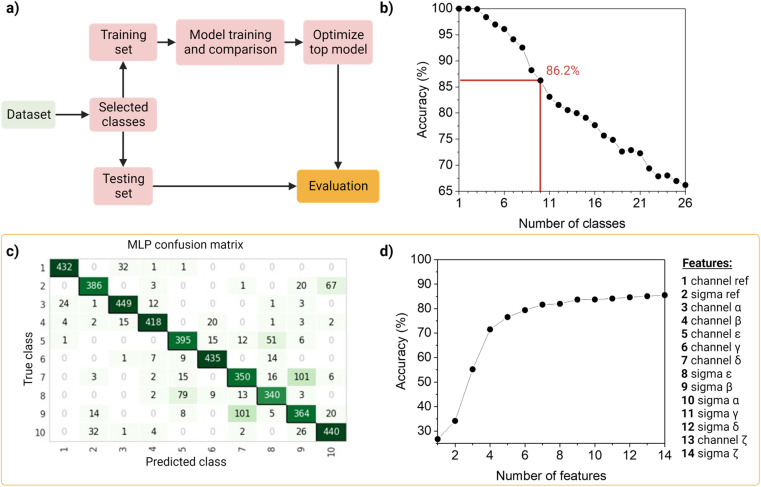
Machine learning barcoding strategy. (a) Model training workflow, the model training and comparison step (together with the training and testing set split) and the top model optimization, were performed 26 times, discarding the least precise each time. (b) Accuracy fluctuation analysis of the machine learning model according to the number of classes. Red lines indicate the chosen model, using 10 barcodes and obtaining an overall accuracy of ∼86%, a sensitivity of ∼83% and a precision of ∼86%. All top-models in each iteration were optimized to report their accuracy. (c) Confusion matrix of barcode identification, using a multi-layer perceptron classifier. The diagonal indicates the number of correct barcode classifications. (d) Accuracy fluctuation analysis according to the number of features, discarding them in order of importance, as shown in the table on the right. The feature importance order was obtained from the second-best performing model (light gradient boosting machine). Feature importance is calculated by the amount that each attribute split point improves the performance measure, weighted by the number of observations the node is responsible for. Non-optimized models were used for this test, thus the difference in the final accuracy shown in the graph.

To identify and classify the barcodes, we developed an automated barcode reader, utilizing machine learning (Fig. 4).

First, as the real data is heterogenous in the amount of samples per class, we wanted the dataset to reflect this as such an unbalanced dataset (that is, the number of observations per class is not equal, Table S2†), containing the 26 barcodes with 14 features (7 intensity values, 7 sigma values from the channels and the merged channel). The dataset was first standardized using *Z*-scores to accommodate variance, and then randomly split into a training set (80%) and a testing set (20%) in a stratified way. The data was used to train and compare fifteen machine-learning models using Pycaret (v2.3.4).^35^ All models were evaluated using a ten-fold cross-validation protocol. We compared a total of fifteen approaches, of which six were linear and nine non-linear (Table S3†). These classifiers were sorted on accuracy (Table S4†), that is, the number of correct predictions divided by the total number of predictions (Table S5†). As a result, the top-three classifiers were: Multi-Layer Perceptron (MLP^31^), Light Gradient Boosting Machine (LGBM^31^), and Random Forest (RF^32^), and their accuracies on the 10-class model, described below, before tuning were ∼83%, ∼80 and ∼79%, respectively. The top classifier (MLP) is a neural network, which are computational models based on the structure of the human brain, where neurons compose the multiple processing layers. To test whether the information extracted from the multi-channel acquisition helped the classification of the barcodes, we repeated the analysis using only the features extracted from the three main acquisitions channels (α, β, δ), which led to a drop in the accuracy of more than 20% (Table S6†). This confirmed the value of this multi-channel strategy to exploit information from “undesired” phenomena such as spectral overlap, and radiative and non-radiative interactions (dye–dye, dye–matrix) without explicitly making a mathematical model for it.

We then investigated the trade-off between the number of classes and the accuracy of the optimized top model (Fig. 4b). On one hand, different applications of our barcode reader may need stricter accuracy, which would be achievable only with fewer classes. On the other hand, some other applications would benefit from having more classes, even if that means a drop in accuracy. Starting with the general model of 26 barcodes, we re-run the computational workflow twenty-five more times, and performed backward class elimination, *i.e.*, we dropped one class at a time (the one with the worst model-based precision (Table S4†) and re-trained the model (Fig. S12†). For each subset of investigated classes, we analyzed the performance of the top algorithms based on accuracy. As expected, decreasing the number of classes improves the performance of the model. The accuracy of the model starts at 100% when evaluating one, two or three classes and decreases almost linearly with the addition of new classes until it reaches 65% when taking all 26 classes. For later implementation in a fluorescent-based assay, one may evaluate which target accuracy is required for the intended application and choose the corresponding number of classes. Therefore, our barcode reader can be customized to the application's needs by lowering or increasing the accuracy to include more or fewer classes.

For further demonstration of the barcode reader, we selected a model with a good trade-off between accuracy and the number of included classes, for a generic application: the 10-class model with an accuracy of 86.24% (Table S7†). The barcodes in our final model consisted of one single color (barcode 1), eight dual colors (barcodes 12–18, barcode 21), and one triple-mixture (barcode 25). The hyperparameters optimized while tuning the model can be found in Table S8,† these were automatically selected by Pycaret when using the *tune_model* function. The resulting confusion matrix is shown in Fig. 4c. Most barcodes are correctly classified: out of the total 10 classes, 6 are classified with a precision of over 80%, while the rest barely go below 70%. For example, barcodes 7 and 9 are the most confused, with a precision of 73% and 69%, respectively (Fig. 4c).

Then, to get more insights into our barcode reader, we performed a feature importance analysis (Fig. 4d). Although we chose an MLP for our application, as it achieved the highest accuracy (>85%), neural networks are a rather opaque model.^48^ Therefore, for this analysis, we took the second-best performing model (LGBM), an improved algorithm of a decision tree, which accuracy is close to that of the neural network (∼80%) and is more interpretable. Feature importance here is calculated by the amount that each attribute split point in the decision tree improves the performance measure, weighted by the number of observations the node is responsible for. The resulting feature importance plot (Fig. S9†) highlights that most of the information is encoded in the intensity values, as the model accuracy is close to saturation before adding the sigma values. The merged values of both intensity and sigma are the most predictive features, followed by the intensity of channels α, β, ε, γ and δ, in this order. In the future, we aim to implement explainable AI approaches that could describe neural networks.^33^ Moreover, we also evaluated the number of observations per class needed to reach the maximum accuracy of the model (Fig. S10†). The results show that 1000 observations (*i.e.*, NPs) are enough to reach the target accuracy of the given dataset. The information obtained from these evaluation tests is relevant to planning and building future datasets based on this system: for instance, by adding an extra fluorophore (color), changing or adding intensity levels, or even reducing screening time.

**Fig. 5:**
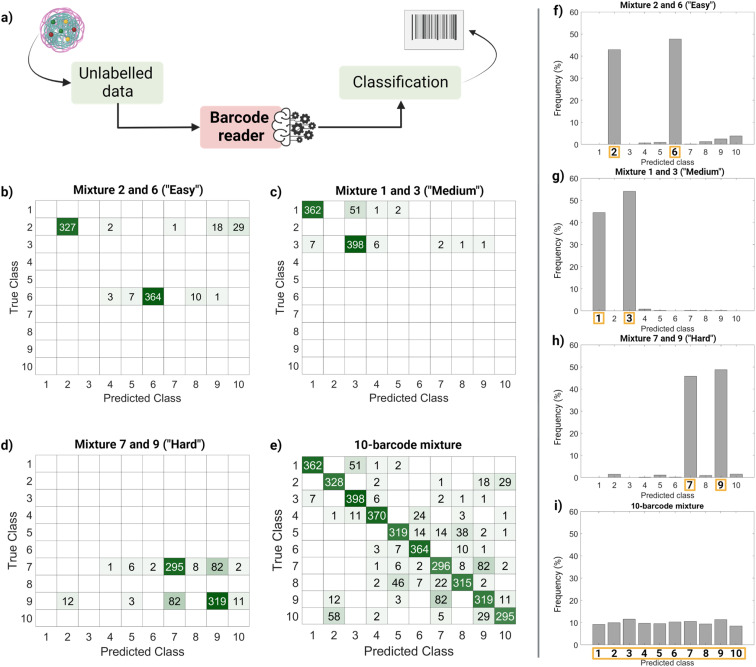
Barcode reader proof-of-concept test on virtually generated mixtures of barcoded nanoparticles (without labels). (a) Barcode reader workflow: we input unlabeled barcodes (from unseen data) into the model to identify them. (b–e) Barcode reader results. Class predictions are represented in a confusion matrix, correct classifications are found in the diagonal and misclassifications in the off-diagonal cells. Different mixtures with distinct levels of difficulty were tested: (b) easy (barcodes 2 and 6), (c) medium (barcodes 1 and 3), (d) hard (barcodes 7 and 9) and (e) all the barcodes together. (f–i) Histograms showing the frequency of class predictions for each mixture. The correct class is highlighted in orange; however, this does not mean the classifications shown in that column are all correct, false positives may be included.

Finally, we performed a ‘real-life’ test of the barcode reader (Fig. 5). Using the confusion matrix in Fig. 4c, we identified which classes were harder to separate (less precise) and easier to separate (more precise). Based on that, we used external data (unseen by the trained model and unlabeled) to make four sets of virtual mixtures, mimicking different real-life situations. The mixtures combined several barcodes in the same ratio according to the following criteria: barcodes that were usually confused and therefore hard to separate (classes 7 and 9), hardly ever confused and therefore easy to separate (classes 2 and 6), found in the average of confusion (classes 1 and 3) and, finally, all of them (classes 1–10). Fig. 5a summarizes the workflow followed for this performance test, Fig. 5b–e display the corresponding confusion matrixes and Fig. 5f–i show the histograms with the frequency of predictions for each class in each mixture. The barcode reader performance varied depending on the mixture, reaching an accuracy of 75% for the ‘harder’ mixture (most confused classes), up to 95% of accuracy for the ‘easier’ mixtures (less confused classes). Overall, the model performed as expected with an average accuracy of 85%.

This output (Fig. 5f–i) can be useful to identify the most frequent hits within a mixture. An example of a setting in which this methodology can be found useful is the field of drug delivery. For instance, candidate nanocarriers with varying surface properties could be evaluated simultaneously in biological systems using this barcode strategy, since the dye is encapsulated, and the surface remains undisturbed.^34^

## Conclusions

Despite the great interest of dye-loaded polymeric nanoparticles as barcodes for biological assays, a limited number of codes are available due to spectral overlap, energy transfer, self-quenching, and the intrinsic heterogeneity of nanoparticles. Some strategies have been successfully exploited to minimize self-quenching and improve encapsulation efficiency of polymeric nanoparticles. In contrast, less attention has been directed to the use of data analysis techniques like machine learning to improve separation of barcodes, or to exploit information related to optical artifacts and ‘undesired’ phenomena such as energy transfer.

In this paper, we presented a workflow to write, read and classify optically barcoded polymeric nanoparticles with un-altered surface properties. This strategy is based on dye-loaded nanoparticles, multi-channel detection and machine learning classification. With the resulting barcode reader, we were able to distinguish all 26 classes with an accuracy of ∼65% and 10 selected classes with an accuracy of ∼85%. Depending on the accuracy required by the specific application of the nano-barcodes, one can choose a barcode reader model with more classes (less accurate but increased throughput) or fewer classes (more accurate but reduced throughput). We observed that heterogeneity between particles remains one of the main factors that compromise the separation of nano-barcodes, which makes differentiation of barcode populations by intensity levels more challenging than separation by colours. Therefore, the proposed barcode scheme could be extended by incorporating more dyes (in the near-UV or far-red) into the pool. There are also a few opportunities to increase accuracy of the classifier, by improving homogeneity of nano-carriers (in size and/or encapsulation), or by measuring other optical properties such as fluorescence life-time, as additional features for the classifier.

Overall, we envision that this strategy can be easily implemented in the current pipeline of several bioassays to increase their throughput, accelerating time-consuming processes by testing different batches simultaneously and reducing the associated costs.

## Experimental

### Materials and reagents

All PLGA-based polymers were purchased from Akina Inc (USA): PLGA polymer with 50 : 50 LA : GA ratio and Mn = 25–35 kDa (#AP082); PLGA–PEG with 50 : 50 LA : GA ratio and *M*_w_ = 30 : 5 kDa (#AK102). Acetonitrile HPLC-grade was used as solvent for the nanoprecipitation process. Cyanine dyes were acquired from Sigma-Aldrich: DiO (#D275), DiI (#D3911), DiD (#D7757). Dye stock solutions were prepared in acetonitrile at a concentration of 1 mM.

### Particle formulation and ensemble characterization

PLGA–PEG nanoparticles were formulated by bulk nanoprecipitation. Succinctly, a 10 mg ml^−1^ polymer mixture (35% PLGA, 65% PLGA–PEG) with variable concentration of dye (1–10 μM) was prepared in acetonitrile (solvent phase). Nanoprecipitation was achieved by adding the polymer solution dropwise to ultrapure water at a ratio solvent:anti-solvent of 1 : 10, under vigorous stirring (700 rpm) at RT. Solvent evaporation was allowed overnight at 400 rpm, RT, protected from light in a fumehood. Particle purification from non-encapsulated dye was performed with a 10 kDa 0.5 ml Amicon-Ultra filter following manufacturer's instructions.

Hydrodynamic radius was measured using Dynamic Light Scattering (DLS) using a Zetasizer Nano-ZS (Malvern Panalytical), with a 633 nm laser and 173° Backscatter detector. Bulk fluorescence was read using a Varian Cary Eclipse Fluorescence Spectrophotometer (Agilent Technologies, USA), with a monochromator that allowed excitation at 488, 552 and 638.

### Single particle characterization: confocal microscopy

For imaging, a 2000× dilution from the nanoparticle stock in PBS 1× was loaded in a 30 μl custom-made flow chamber, consisting of a glass microscope slide (Menzel Gläser, 76 × 26 mm, thickness 1 mm) and a coverslip (Menzel Gläser, no. 1.5, 24 × 24 mm, thickness 170 μm). Single nanoparticles were imaged using a Leica HC PL APO 100×/1.4 oil-immersion objective with a Leica DMi8 microscope with a confocal TCS SP8 unit. Each sample was excited sequentially with a 488, 552 and 638 lasers at 0.5% of power, and the signal was recorded with a hybrid detector Leica hyD1. The corresponding 6 channels (Table 1) were defined based on spectral properties of the three selected dyes: the three main channels (α, β, δ) were defined by combining the optimal laser line for each fluorophore with the corresponding emission window – α (DiD, 638 ex., 648–750 em.), β (DiI, 552 ex. 563–620 em.) and δ (DiO, 488 ex., 498–538 em.) – and the three additional channels (γ, ε, ζ) were proposed by pairing the excitation line of one fluorophore with the emission window of other (as long as the emission λ was longer than the excitation), in order to collect information related to spectral overlap, dye interactions, *etc.*

**Table 1:** Channel optical specifications

Channel	Laser line (nm)	Detection window (nm)
α	638	648–750
β	552	563–620
γ	552	648–750
δ	488	498–538
ε	488	562–620
ζ	488	648–750

Each 16 bit image in the 6-channel sequence had a size of 504 × 504 pixels, a pixel size of 92.45 nm in *X* and *Y*, and was acquired with a scan speed of 400 Hz lines per second (0.8 frames per second), 16-line average and 1.0 airy unit.

Raw images were processed with ImageJ (NIH, USA) software and ThunderSTORM plug-in.^30^ First, the six images of each sequence were merged into a reference image and ThunderSTORM was used to find the centroid of each point spread function (coordinates *x*,*y*) and extract the Gaussian-fitted intensity and sigma.

Then, with MATLAB R2019a (MathWorks), each PSF/localization on the reference image was matched to the corresponding localization in the individual channels using their coordinates (shortest Euclidean distance in a radius of 120 nm). The resulting report includes 7 values of intensities and 7 values of sigma (6 channels + reference channel) for each observation (PSF/localization), a total of 14 features. For each barcode or class, 10–15 fields of view (FOVs) were analyzed to build our dataset (with a minimum number of 2000 observations (particles) per class (barcode)).

### Optical fingerprint visualization: principal component analysis (PCA)

To visualize 14-D data, principal component analysis (PCA) was used to reduce the dimensionality of our data and investigate possible patterns in a 2D and 3D space. This analysis was run with MATLAB R2021a's ‘*pca*’ function, using the Singular Value Decomposition (SVD) algorithm to center the data, and a matrix containing the 14-feature values of at least 2000 observations from each barcode or ‘class’ as input argument. As a result, the function returns the coefficient matrix (where columns correspond to the coefficients for one principal component in descending order of component variance), the principal components scores, and the principal components variances. Using the scores for each of the top 3 principal components, we were able to plot the PCA figures.

### Machine learning classification and barcode reader

#### Preprocessing

The original unbalanced dataset, consisting of 14 features (6 intensity values, 6 sigma values, and the corresponding merged values), was standardized using *Z*-scores prior to training. The datasets were split into a training set (80%) and a testing set (20%) in a stratified way, to maintain the original unbalance of the classes.

#### Algorithms and training

PyCaret (v2.3.4)^35^ was used to compare models built-in in Sci-kit Learn (v0.24.2), select the best performing one, and optimize it. The models trained and compared during this process were: multi-layer perceptron classifier,^36^ light gradient boosting machine,^31^ random forest classifier,^32^ extra trees classifier,^37^ support vector machine^38^ (both radial and linear kernel), logistic regression,^39^ K-neighbors classifier,^40^ decision tree classifier,^41,42^ linear discriminant analysis,^43^ ridge classifier,^44^ naive bayes,^45^ quadratic discriminant analysis^46^ and Ada boost classifier.^47^ A total of 26 different sub-sets from the original dataset were used, excluding each time the least precise class, after model training.

#### Model validation

After selecting the model with the optimal number of classes, it was optimized and evaluated. The optimization process was focused on increasing the accuracy of the model; the metrics optimized were doubling the size of the hidden layers in the MLP classifier and changing the learning rate from ‘constant’ to ‘adaptive’ (Fig. S12b and c†). This tuning process is done automatically by calling the function “*tune_model*” in Pycaret, which by a process of trial and error in 10 iterations, adapts hyperparameters to obtain a better performance. The model was validated with ten-fold cross-validation. To evaluate the performance of the algorithm, metrics based on the confusion matrix were exploited: accuracy (Table S4†) and precision (Table S4†). And the final model was chosen based on its accuracy.

Furthermore, the lack of chance correlation was verified using Y-scrambling (Fig. S11†), this analysis is performed by randomly shuffling the target column of the dataset while keeping the input features unchanged, so the features do not match their label anymore, and retraining the model and noting its accuracy. This procedure is repeated 100 times.

### Data visualization and graphics

MATLAB R2021a (MathWorks) and Origin 2020 (OriginLab Corporation, Northampton, MA, USA) were used for data visualization. Schematic figures were created with https://BioRender.com.

## Data availability

The codes and datasets used for this project are publicly available at: https://github.com/n4nlab/BarcodedNanoparticles.

## Conflicts of interest

There are no conflicts to declare.

## Supplementary Material

NA-005-D2NA00648K-s001
