# Supplementary material for: Identification of fluorescently-barcoded nanoparticles using machine learning
Source: Nanoscale Adv. 2023 Mar 23;5(8):2307–17. doi: 10.1039/d2na00648k (PMC10089084; doi:10.1039/d2na00648k)
Supplement: NA-005-D2NA00648K-s001 [file NA-005-D2NA00648K-s001.pdf]

Electronic Supplementary Material (ESI) for Nanoscale Advances.
This journal is © The Royal Society of Chemistry 2023

Identification of fluorescently-barcoded nanoparticles using machine learning

Ana Ortiz-Perez *[1], Cristina Izquierdo-Lozano *[1], Rens Meijers[1], Francesca Grisoni[1], Lorenzo Albertazzi[1]

[1]Institute for Complex Molecular Systems (ICMS), Eindhoven University of technology, PO Box 513, 5600 MB Eindhoven, The Netherlands

List of figures and tables

PART 1 Characterization of dye encapsulation

- **Fig. S1.** Dye encapsulation and fluorescence emission evaluation with bulk spectrophotometry.
- **Fig. S2.** TIRF images of single DiI-loaded nanoparticles with 1, 10, 75 and 100 uM of dye.
- **Fig. S3.** Influence of DiO concentration in nanoparticle properties.

PART 2 Bulk physicochemical characterization of barcodes

- **Table S1.** Barcode bulk physicochemical characterization by Dynamic Light Scattering (DLS) and Nanoparticle Tracking Analysis (NTA).
- **Fig. S4.** Barcode bulk physicochemical characterization. by Dynamic Light Scattering (DLS) and Nanoparticle Tracking Analysis (NTA).
- **Fig. S5.** Bulk emission spectra of 26 barcodes excited with 488, 552 and 638 laser lines.

PART 3 Single Nanoparticle optical characterization with Confocal Microscopy

- **Fig. S6.** Single nanoparticle optical characterization with confocal microscopy.
- **Fig. S7.** Comparison between bulk and single-particle fluorescence intensities for two intensity levels (1, 10 μM) of the same color (DiI).

PART 4 Principal Components Analysis (PCA)

- **Fig. S8.** Different perspectives of the PCA plot for barcodes 1-26.

PART 5 Machine learning

- **Table S2.** Overview of the unbalance in the dataset.
- **Table S3.** Classifiers trained and compared in the creation of the supervised machine-learning model.
- **Table S4.** Metrics used to assess the models and their formulas.
- **Table S5.** Scores of the different classifiers trained with the full dataset of 26 barcodes, sorted by higher accuracy first.
- **Table S6.** Scores of the different classifiers trained with the full dataset of 26 barcodes, sorted by higher accuracy first, using only the features extracted from the three main acquisitions channels (α, β, δ).
- **Table S7.** Scores of the different classifiers trained with the 10-class model.
- **Fig. S9.** Feature importance plot of the second-best performing model, a Light Gradient Boosting Machine.
- **Fig. S10.** Accuracy according to dataset size.
- **Table S8.** Top-performance model, MLP classifier, hyperparameters before and after optimization.
- **Fig. S11.** y-scrambling analysis of the 10-class model.
- **Fig. S12.** Metrics per class for each of the 26 models trained to study the trade-off between accuracy and number of classes.
- **Table S9.** Model stability analysis.

PART 1 Characterization of dye encapsulation

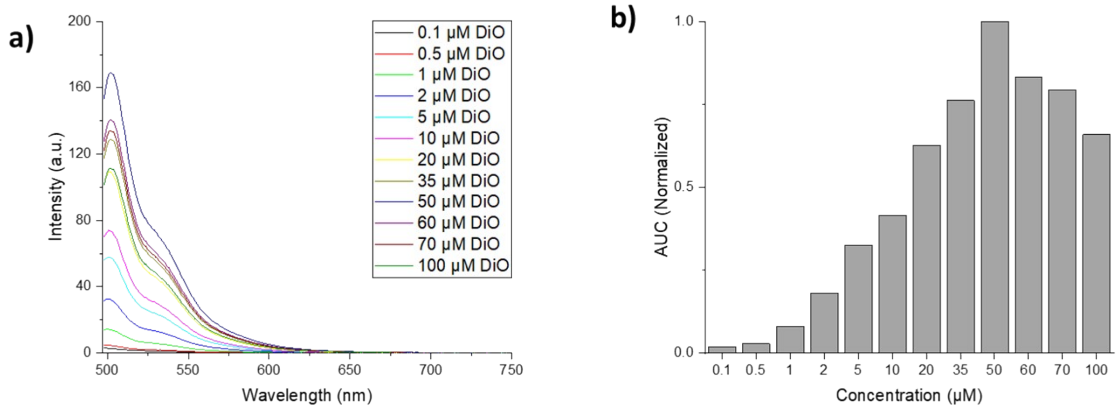

Fig. S1. Dye encapsulation and fluorescence emission evaluation with bulk spectrophotometry (a) DiO spectra of nanoparticles with concentrations ranging from 0.1 µM to 100 µM of DiO, (b) Quantification of fluorescence spectra as normalized area under the curve (AUC) of nanoparticles with concentration ranging from 0.1 µM to 100 µM of DiO.

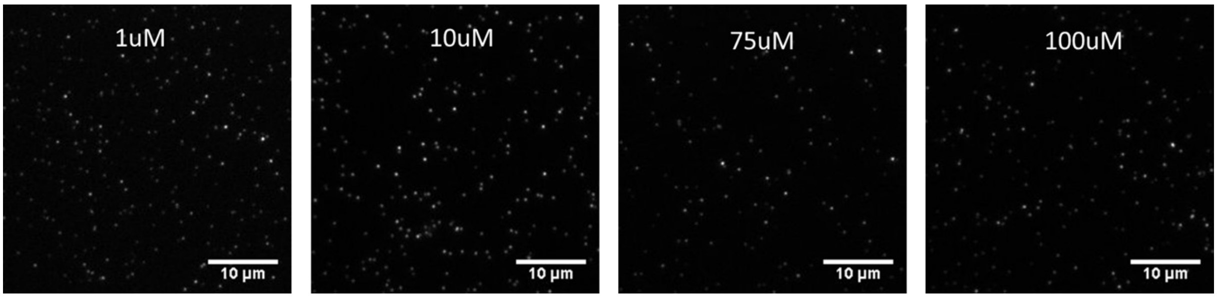

Fig. S2. Total internal reflection fluorescence (TIRF) images of single DiI-loaded nanoparticles with 1, 10, 75 and 100 µM of dye. Optical parameters: 100x, 100 ms, 561 nm excitation at 2% laser power, TIRF angle 3930,0.

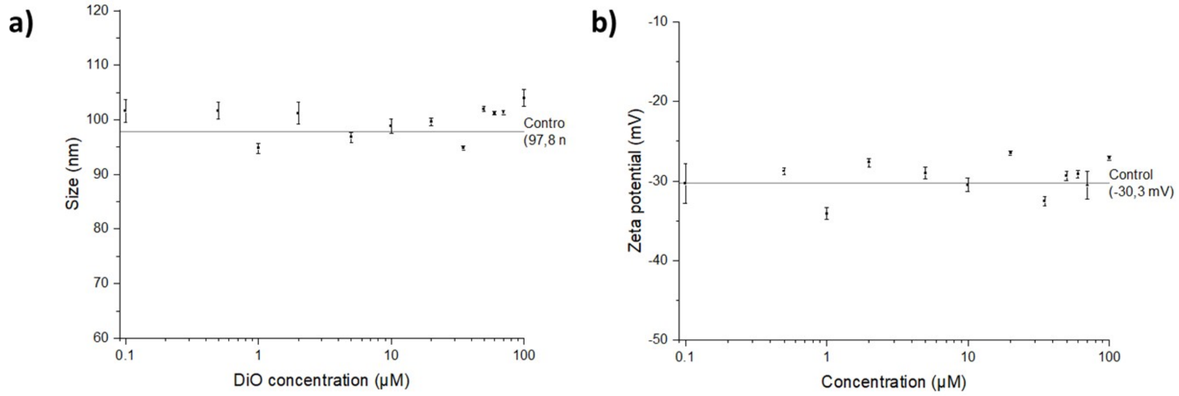

Fig S3. Influence of DiO concentration in nanoparticle properties: (a) size measured as hydrodynamic radius (Dynamic light scattering (DLS)) and (b) Z-potential. Three independent measurements, error bars displayed as standard deviation.

PART 2 Bulk physicochemical characterization of barcodes

Table S1. Barcode bulk physicochemical characterization by Dynamic Light Scattering (DLS) and Nanoparticle Tracking Analysis (NTA).

	Zetasizer characterization/ DLS				Nanosight Characterization/ NTA			
**Barcode**	**Z-average (nm)**	**SD**	**PDI**	**SD**	**Size (nm)**	**SD**	**NPs/ml $*10^9$**	**SE $*10^9$**
**1**	81.78	0.49	0.08	0.01	99	18.9	1.24	0.0259
**2**	85.45	0.49	0.08	0.01	102.4	28.3	2.51	0.0501
**3**	79.93	0.40	0.07	0.01	99.5	18.2	1.72	0.0410
**4**	83.37	0.43	0.06	0.01	100.5	20.2	1.98	0.0663
**5**	79.98	0.71	0.07	0.02	97.2	18.7	1.65	0.035
**6**	82.43	1.05	0.07	0.02	102.7	29	1.48	0.0612
**7**	77.24	0.28	0.05	0.02	98.9	19.2	1.45	0.0231
**8**	80.98	0.52	0.07	0.01	99	25.4	2.21	0.0264
**9**	78.20	0.79	0.07	0.01	97.9	20.7	1.67	0.04
**10**	80.60	0.70	0.06	0.02	98.5	24.0	1.99	0.0173
**11**	81.66	0.88	0.07	0.02	99.2	15.7	1.64	0.822
**12**	85.13	0.50	0.07	0.01	102.7	21.4	1.91	0.0483
**13**	81.55	3.27	0.06	0.03	101.2	17.8	1.42	0.0277
**14**	86.94	0.46	0.06	0.02	107	20.0	1.18	0.069
**15**	82.35	0.40	0.08	0.01	100.4	18.2	1.87	0.0843
**16**	85.11	1.04	0.07	0.02	100.9	20.3	1.92	0.0267
**17**	82.87	0.59	0.06	0.01	103.9	16.7	1.89	0.0112
**18**	87.68	0.95	0.05	0.02	99.4	19.2	2	0.0144
**19**	81.83	0.53	0.08	0.01	98.9	16.2	2.31	0.0455
**20**	84.05	0.28	0.06	0.02	98.6	15.8	1.75	0.0421
**21**	85.56	0.67	0.07	0.02	102.1	23.2	1.42	0.0144
**22**	87.96	0.87	0.06	0.02	101.3	17.7	1.52	0.0269
**23**	86.53	0.57	0.07	0.01	102.8	20	1.45	0.0241
**24**	86.57	1.30	0.07	0.02	102.7	17.7	1.55	0.0593
**25**	88.15	0.63	0.06	0.01	107.3	31.2	1.39	0.0284
**26**	91.72	1.25	0.08	0.02	103.5	19.0	2	0.0422

PDI= poly dispersity index, NPs = Nanoparticles, SD= Standard Deviation, SE = Standard Error of the mean

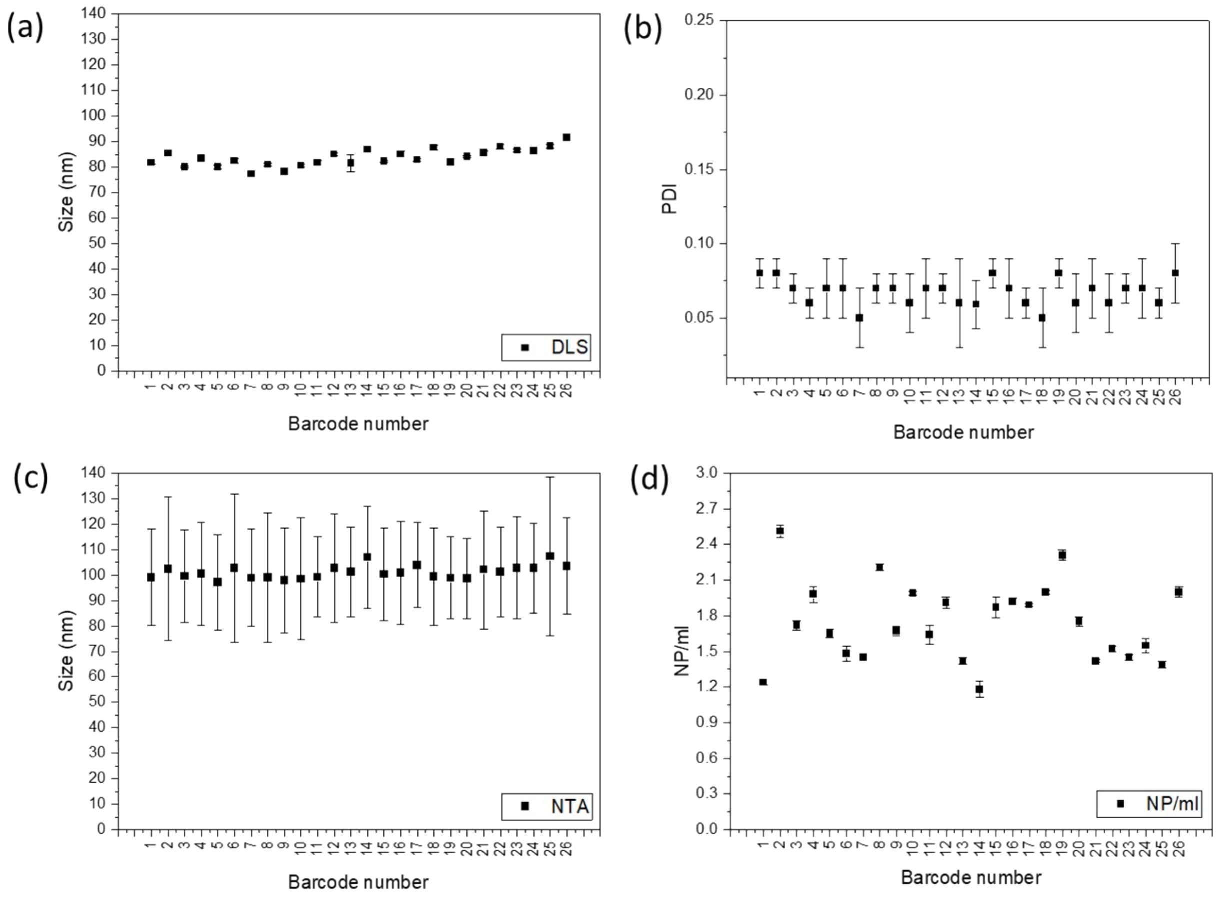

Fig S4. Barcode bulk physicochemical characterization by Dynamic Light Scattering (DLS) and Nanoparticle Tracking Analysis (NTA). (a) Mean diameter of 26 barcodes and DLS. (b) Polydispersity index (PDI) measured by DLS. Average of three measurements, error bars displayed as standard deviation (SD). (c) Mean diameter of 26 barcodes and (d) number concentration (NP/ml) estimated by NTA. Average of three measurements, errors displayed as standard deviation (SD) for the size and standard error of the mean (SE) for the number concentration .

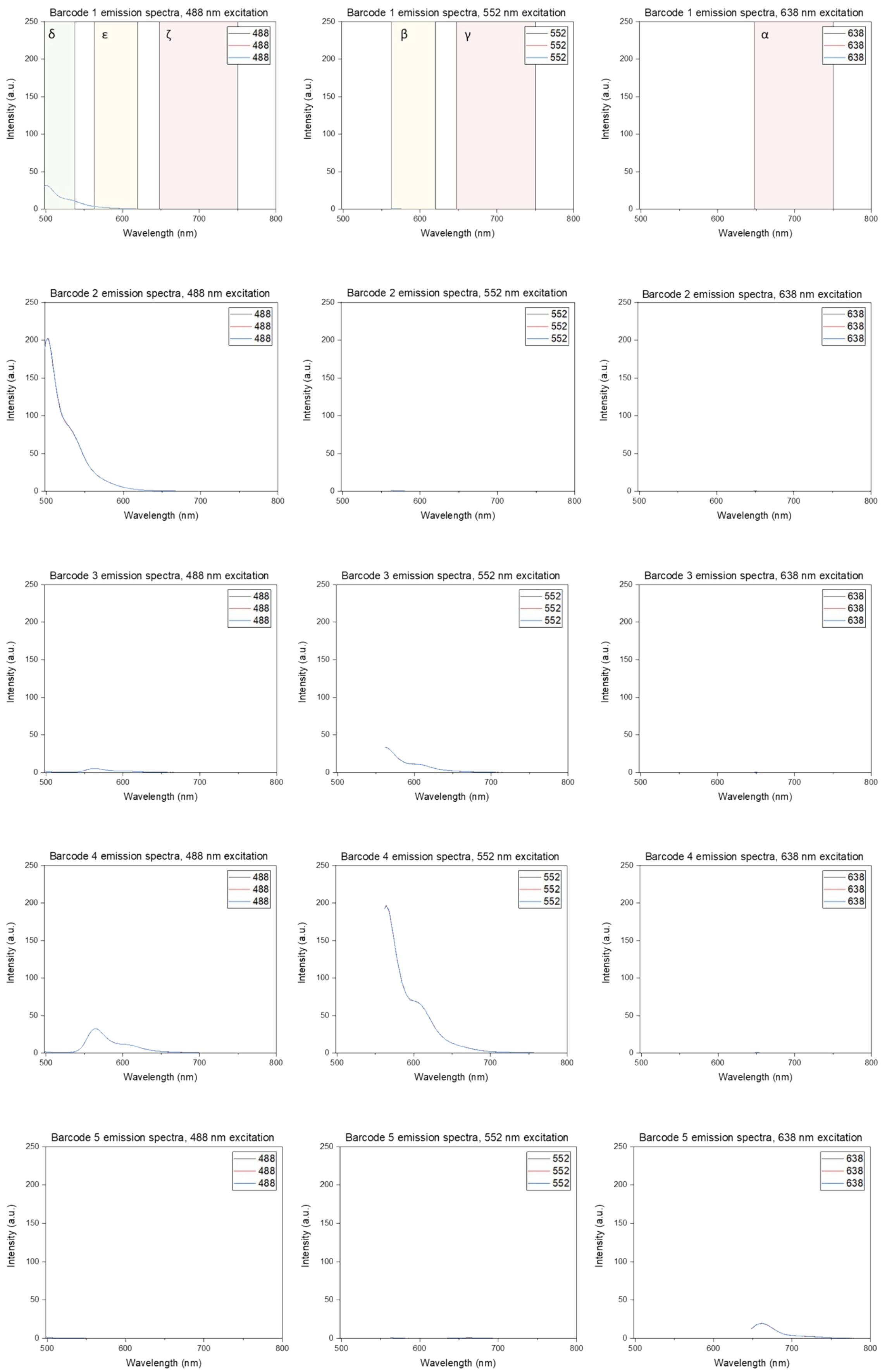
Barcode 1 emission spectra, 488 nm excitation
δ
ε
ζ
Barcode 1 emission spectra, 552 nm excitation
β
γ
Barcode 1 emission spectra, 638 nm excitation
α
Barcode 2 emission spectra, 488 nm excitation
Barcode 2 emission spectra, 552 nm excitation
Barcode 2 emission spectra, 638 nm excitation
Barcode 3 emission spectra, 488 nm excitation
Barcode 3 emission spectra, 552 nm excitation
Barcode 3 emission spectra, 638 nm excitation
Barcode 4 emission spectra, 488 nm excitation
Barcode 4 emission spectra, 552 nm excitation
Barcode 4 emission spectra, 638 nm excitation
Barcode 5 emission spectra, 488 nm excitation
Barcode 5 emission spectra, 552 nm excitation
Barcode 5 emission spectra, 638 nm excitation
488
552
638
Intensity (a.u.)
Wavelength (nm)
0
50
100
150
200
250
500
600
700
800

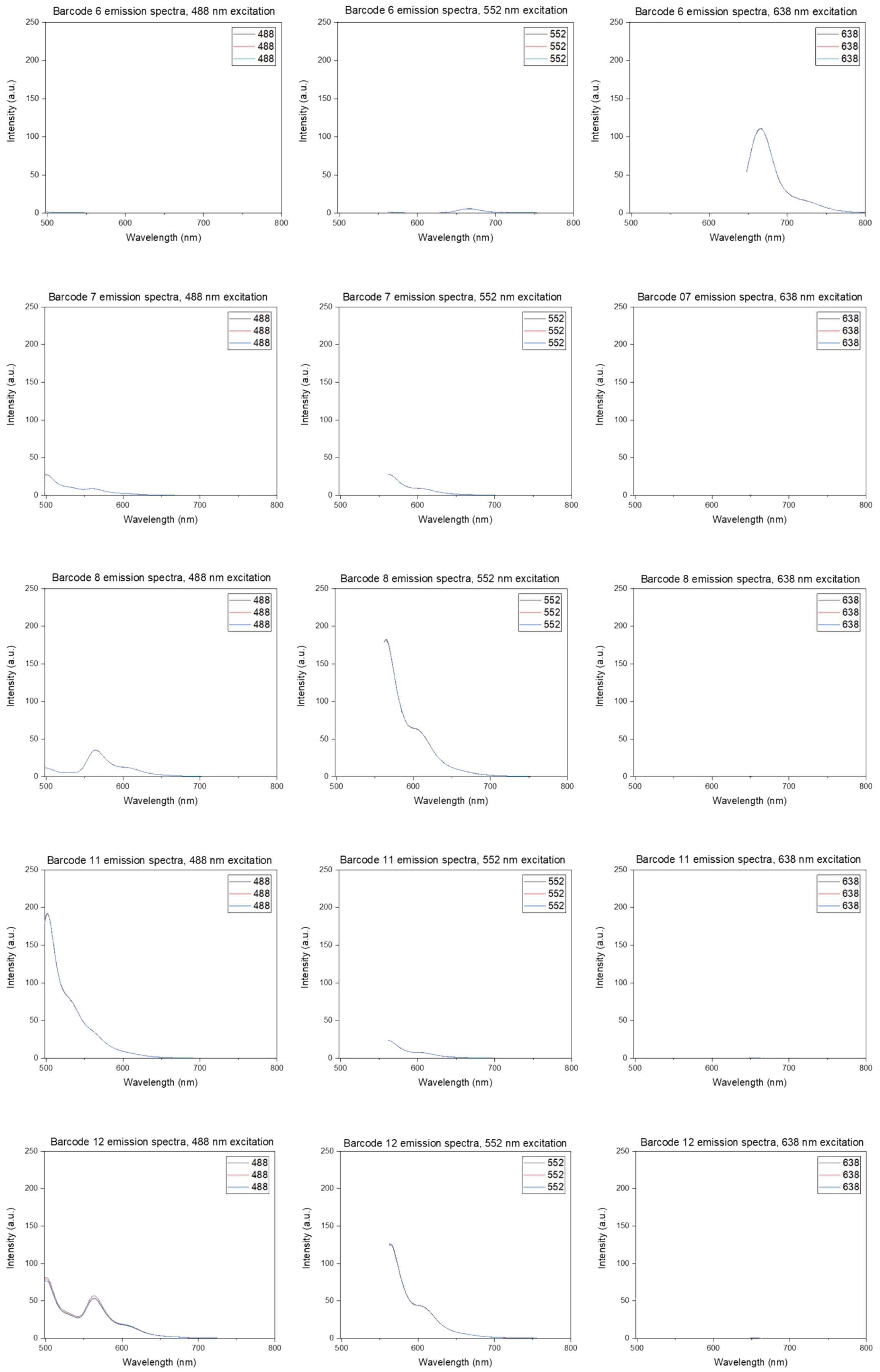
Barcode 6 emission spectra, 488 nm excitation
Barcode 6 emission spectra, 552 nm excitation
Barcode 6 emission spectra, 638 nm excitation
Barcode 7 emission spectra, 488 nm excitation
Barcode 7 emission spectra, 552 nm excitation
Barcode 07 emission spectra, 638 nm excitation
Barcode 8 emission spectra, 488 nm excitation
Barcode 8 emission spectra, 552 nm excitation
Barcode 8 emission spectra, 638 nm excitation
Barcode 11 emission spectra, 488 nm excitation
Barcode 11 emission spectra, 552 nm excitation
Barcode 11 emission spectra, 638 nm excitation
Barcode 12 emission spectra, 488 nm excitation
Barcode 12 emission spectra, 552 nm excitation
Barcode 12 emission spectra, 638 nm excitation
Intensity (a.u.)
Wavelength (nm)

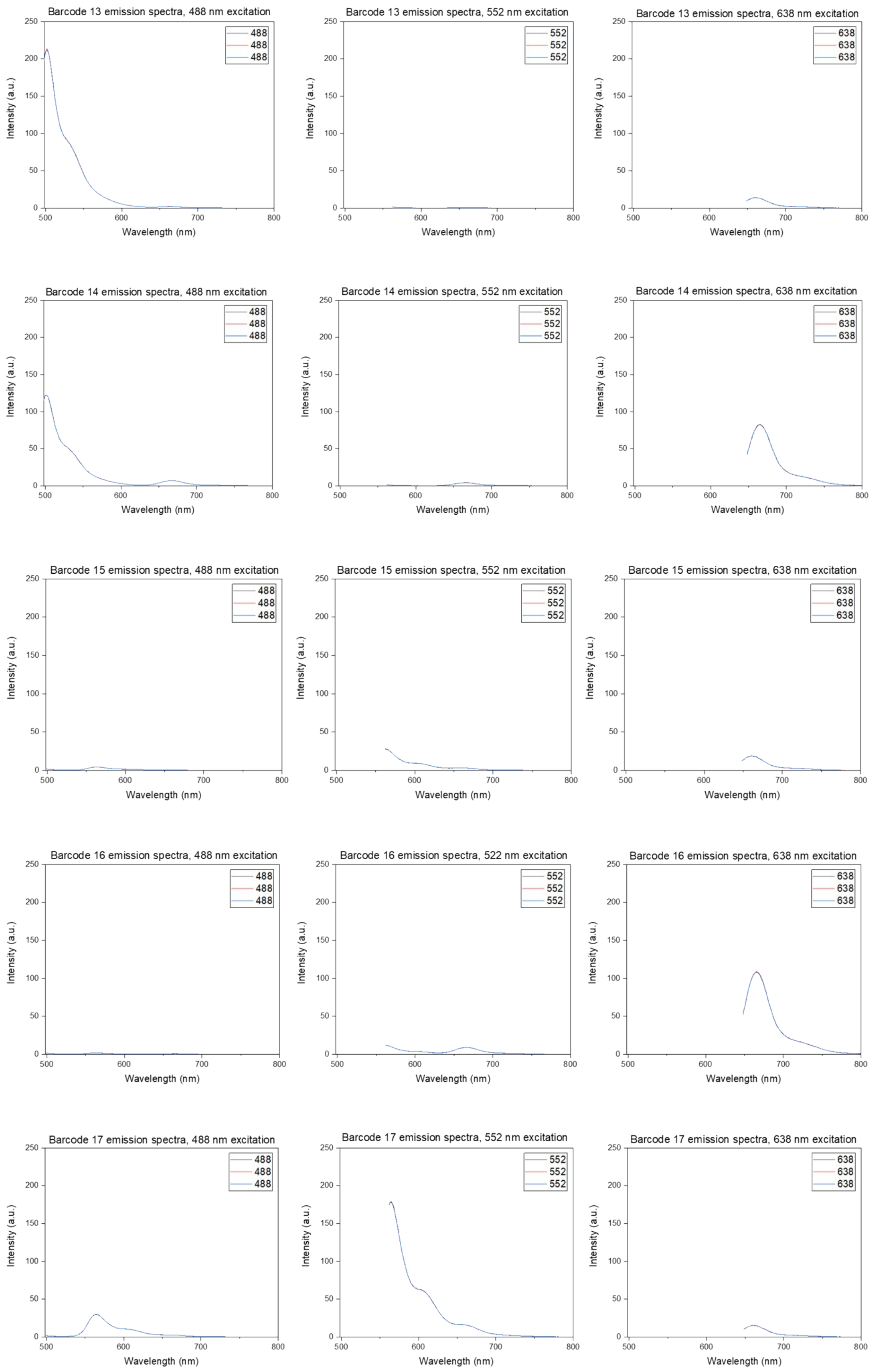
Barcode 13 emission spectra, 488 nm excitation
Barcode 13 emission spectra, 552 nm excitation
Barcode 13 emission spectra, 638 nm excitation
Barcode 14 emission spectra, 488 nm excitation
Barcode 14 emission spectra, 552 nm excitation
Barcode 14 emission spectra, 638 nm excitation
Barcode 15 emission spectra, 488 nm excitation
Barcode 15 emission spectra, 552 nm excitation
Barcode 15 emission spectra, 638 nm excitation
Barcode 16 emission spectra, 488 nm excitation
Barcode 16 emission spectra, 522 nm excitation
Barcode 16 emission spectra, 638 nm excitation
Barcode 17 emission spectra, 488 nm excitation
Barcode 17 emission spectra, 552 nm excitation
Barcode 17 emission spectra, 638 nm excitation
488
552
638
Intensity (a.u.)
Wavelength (nm)
0
50
100
150
200
250
500
600
700
800

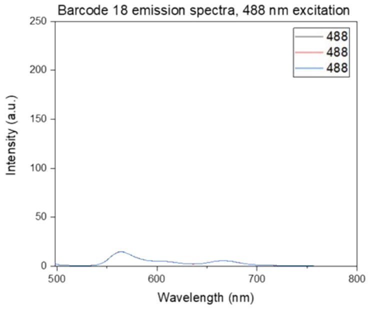
Barcode 18 emission spectra, 488 nm excitation
488
488
488
Intensity (a.u.)
Wavelength (nm)

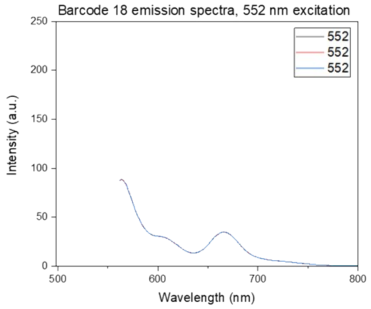
Barcode 18 emission spectra, 552 nm excitation
552
552
552
Intensity (a.u.)
Wavelength (nm)

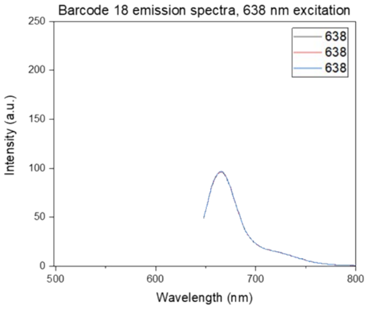
Barcode 18 emission spectra, 638 nm excitation
638
638
638
Intensity (a.u.)
Wavelength (nm)

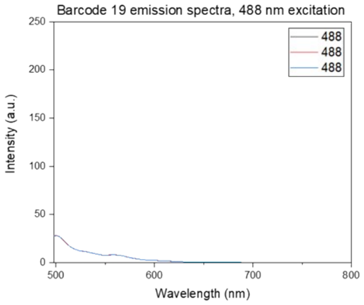
Barcode 19 emission spectra, 488 nm excitation
488
488
488
Intensity (a.u.)
Wavelength (nm)

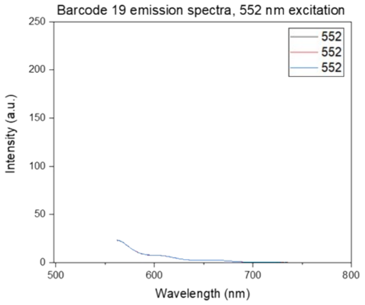
Barcode 19 emission spectra, 552 nm excitation
552
552
552
Intensity (a.u.)
Wavelength (nm)

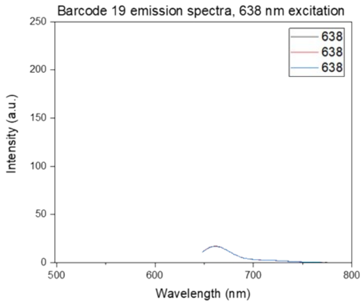
Barcode 19 emission spectra, 638 nm excitation
638
638
638
Intensity (a.u.)
Wavelength (nm)

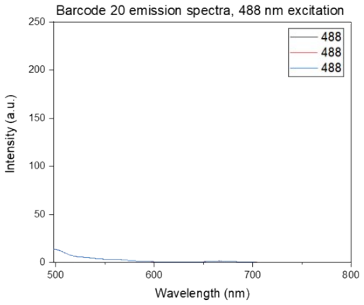
Barcode 20 emission spectra, 488 nm excitation
488
488
488
Intensity (a.u.)
Wavelength (nm)

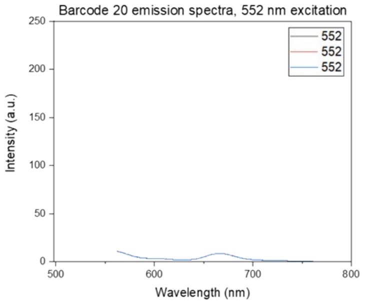
Barcode 20 emission spectra, 552 nm excitation
552
552
552
Intensity (a.u.)
Wavelength (nm)

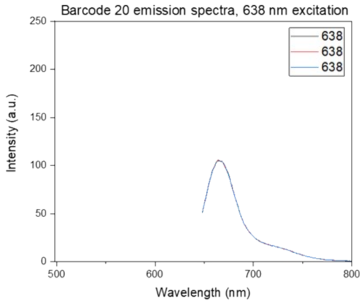
Barcode 20 emission spectra, 638 nm excitation
638
638
638
Intensity (a.u.)
Wavelength (nm)

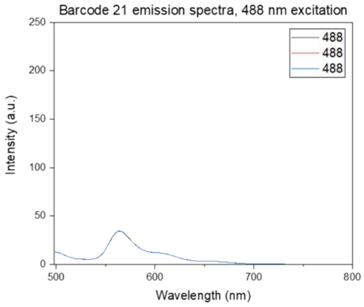
Barcode 21 emission spectra, 488 nm excitation
488
488
488
Intensity (a.u.)
Wavelength (nm)

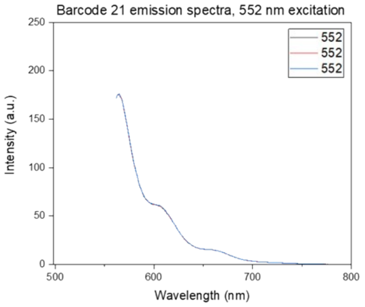
Barcode 21 emission spectra, 552 nm excitation
552
552
552
Intensity (a.u.)
Wavelength (nm)

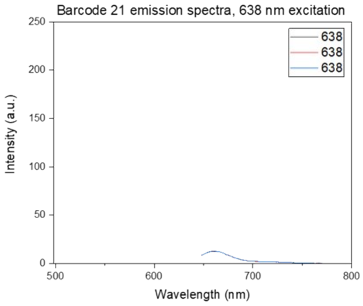
Barcode 21 emission spectra, 638 nm excitation
638
638
638
Intensity (a.u.)
Wavelength (nm)

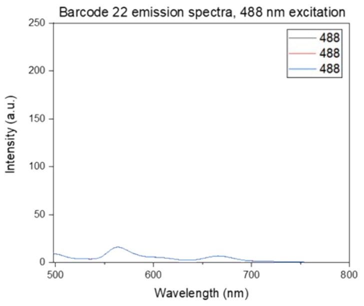
Barcode 22 emission spectra, 488 nm excitation
488
488
488
Intensity (a.u.)
Wavelength (nm)

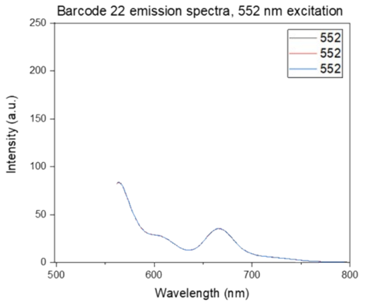
Barcode 22 emission spectra, 552 nm excitation
552
552
552
Intensity (a.u.)
Wavelength (nm)

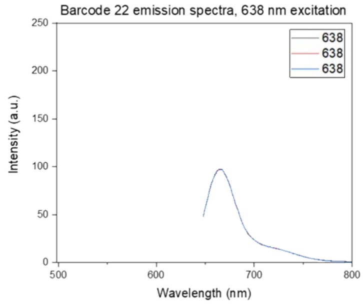
Barcode 22 emission spectra, 638 nm excitation
638
638
638
Intensity (a.u.)
Wavelength (nm)

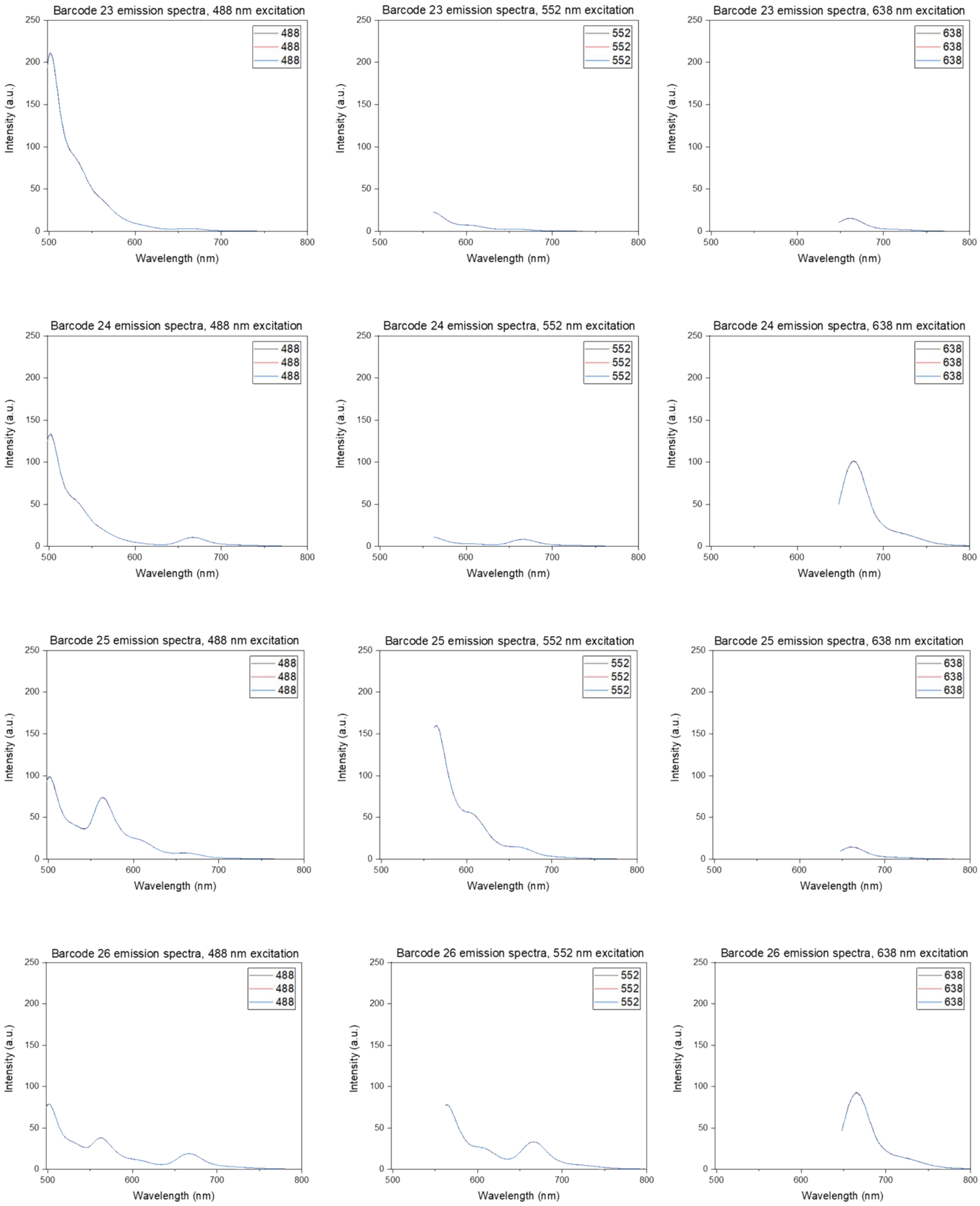

Fig S5. Bulk fluorescence emission spectra of 26 barcodes excited with 488, 552 and 638 nm laser lines, emission collected from 498-750 nm, 562-750 nm and 648-750 nm.

PART 3 Single Nanoparticle optical characterization with Confocal Microscopy

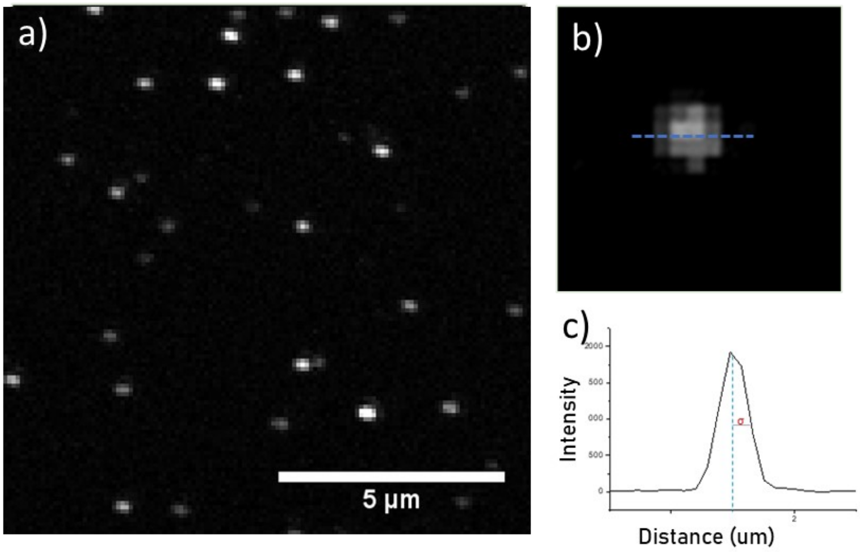

Fig S6. Single nanoparticle optical characterization with confocal microscopy. (a) DiI loaded Poly Lactic-co-glycol Acid – Poly ethylene glycol PLGA-PEG nanoparticles physiosorbed on a glass surface at a medium to low density, (b) zoom in of one point spread function (nanoparticle) and (c) corresponding intensity profile displaying the classical gaussian-shape that is characteristic of a point spread function.

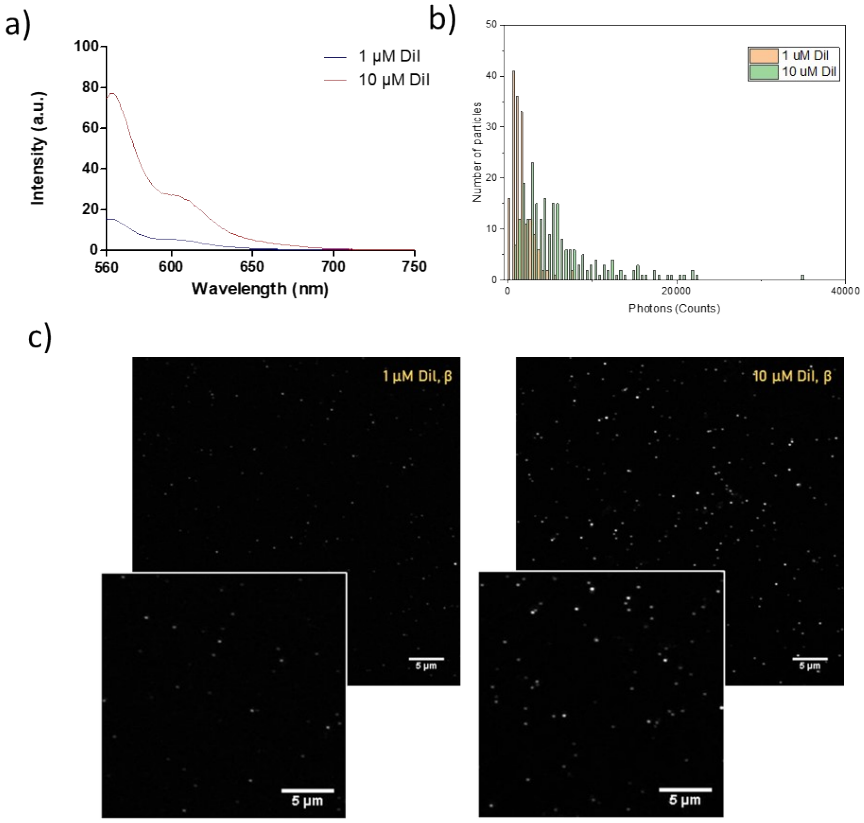

Fig. S7. Comparison between bulk and single-particle fluorescence intensities for two intensity levels (1, 10 µM) of the same color (DiI). (a) Fluorescence spectra of 1 and 10 µM DiI particles measured with (bulk) spectrophotometry. (b) Distribution of fluorescence intensities of 1 and 10 µM particles quantified from (c) confocal images.

PART 4 Principal Component Analysis (PCA)

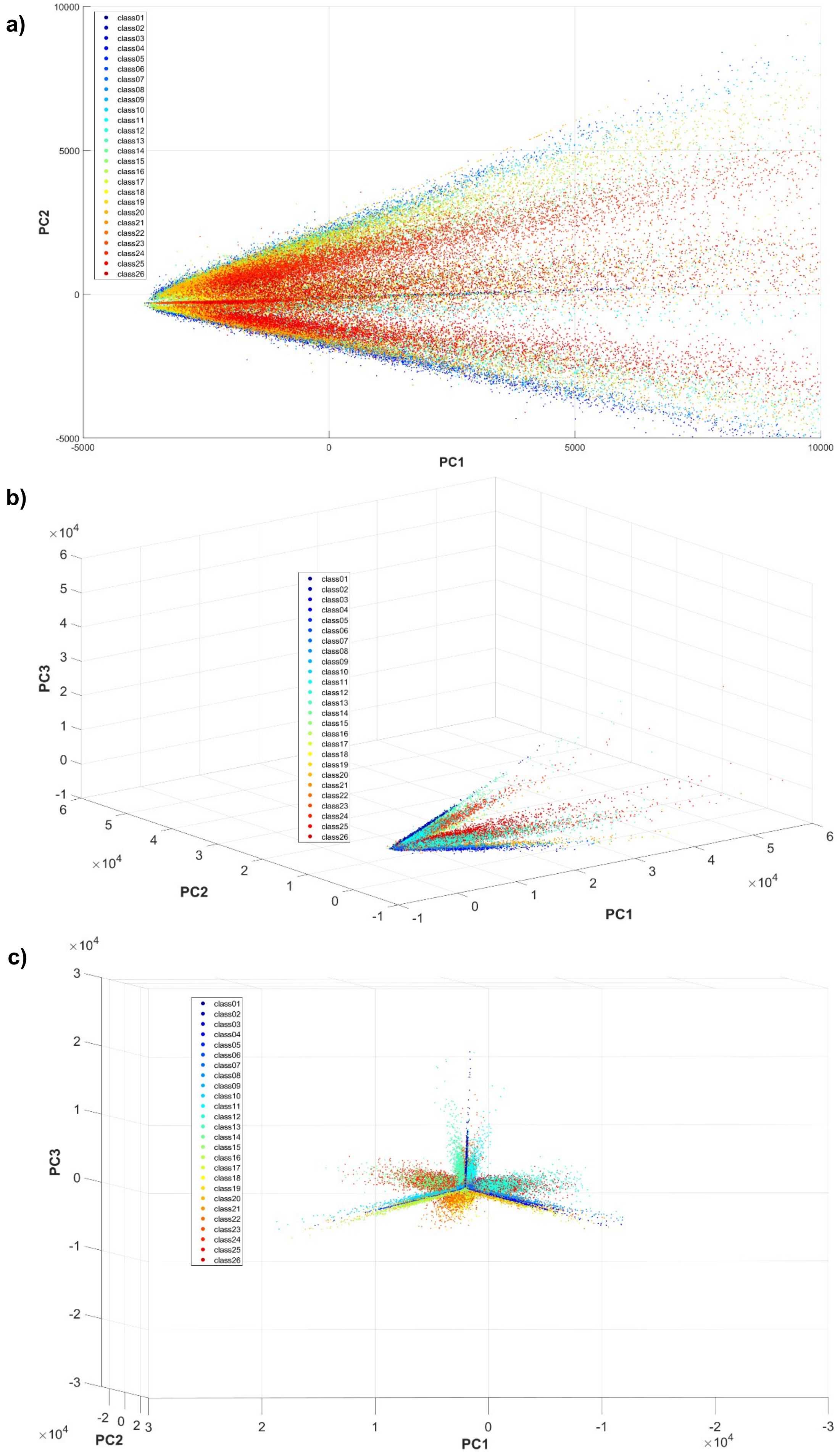

Fig. S8. Different perspectives of the PCA plot for barcodes 1-26. The interactive MATLAB figure can be found in https://github.com/n4nlab/BarcodedNanoparticles. Explained variances for each principal component (PC): PC1 = 80.4087%, PC2 = 13.0798% and PC3 = 5.6811%. (a) 2D plot, (b) 3D plot, side view. (c) 3D plot, front view.

PART 5 Machine learning

Table S2. Overview of the unbalance in the dataset.

Barcode (class)	Samples	Barcode (class)	Samples
1	2902	14	4356
2	4704	15	2488
3	3266	16	7658
4	3700	17	3851
5	1458	18	3195
6	2768	19	2078
7	2091	20	4984
8	3612	21	4213
9	2139	22	3955
10	3424	23	4074
11	3797	24	9784
12	3817	25	6876
13	4906	26	5102

Table S3. Classifiers trained and compared in the creation of the supervised machine-learning model.

Classifier	Abbreviation	Type
**Logistic Regression**	lr	Linear
**K Neighbors Classifier**	knn	Non-linear
**Naive Bayes**	nb	Linear
**Decision Tree Classifier**	dt	Non-linear
**SVM - Linear Kernel**	svm	Linear
**SVM - Radial Kernel**	rbsvm	Linear
**MLP Classifier**	mlp	Non-linear
**Ridge Classifier**	ridge	Linear
**Random Forest Classifier**	rf	Non-linear
**Quadratic Discriminant Analysis**	qda	Non-linear
**Ada Boost Classifier**	ada	Non-linear
**Gradient Boosting Classifier**	gbc	Non-linear
**Linear Discriminant Analysis**	lda	Linear
**Extra Trees Classifier**	et	Non-linear
**Light Gradient Boosting Machine**	lightgbm	Non-linear

Table S4. Metrics used to assess the models and their formulas. Notations: n = number of samples; G = number of classes; n_g = number of samples belonging to the g-th class; n'_g = number of samples predicted in the g-th class; c_{gg} = number of correctly classified samples; c_{gk} = number of samples belonging to class g and predicted as belonging to class k.

Metric	Formula
**Accuracy**	$\frac{\sum_{g=1}^{G} c_{gg}}{n}$
**Balanced accuracy**	$\frac{\sum_{g=1}^{G} \frac{c_{gg}}{n_g}}{G}$
**Sensitivity**	$\sum_{g=1}^{G} \frac{c_{gg}}{n_g}$
**Average Precision**	$\frac{\sum_{g=1}^{G} \frac{c_{gg}}{n'_g}}{G}$
**Precision**	$\frac{c_{gg}}{n'_g}$
**F1 score**	$2 \times \frac{Precision \times Sensitivity}{Precision + Sensitivity}$
**Matthew's Correlation Coefficient (MCC)**	$\frac{\sum_{g=1}^{G}\sum_{k=1}^{G}\sum_{m=1}^{G}(c_{gg} \cdot c_{km} - c_{gk} \cdot c_{mg})}{\sqrt{\sum_{g=1}^{G}\left[(\sum_{k=1}^{G} c_{gk}) \cdot (\sum_{f=1, f \neq g}^{G}\sum_{m=1}^{G} c_{fm})\right]} \cdot \sqrt{\sum_{g=1}^{G}\left[(\sum_{k=1}^{G} c_{kg}) \cdot (\sum_{f=1, f \neq g}^{G}\sum_{m=1}^{G} c_{fm})\right]}}$

Table S5. Scores of the different classifiers trained with the full dataset of 26 barcodes, sorted by higher accuracy first. TT = Total Time. Pycaret's automatic output.

Model	Accuracy	Balanced accuracy	Average Precision	F1	MCC	TT (s)
**MLP Classifier (mlp)**	0.6429	0.6021	0.6501	0.6346	0.6262	11.5060
**Random Forest Classifier (rf)**	0.5983	0.5548	0.5950	0.5872	0.5742	1.5420
**Extra Trees Classifier (et)**	0.5808	0.5398	0.5819	0.5726	0.5607	0.9150
**Gradient Boosting Classifier (gbc)**	0.5785	0.5331	0.5835	0.5675	0.5586	56.3090
**Light Gradient Boosting Machine (lightgbm)**	0.5708	0.5311	0.5716	0.5631	0.5501	2.3770
**SVM – radial kernel (rbfsvm)**	0.5643	0.5096	0.6218	0.5496	0.5466	36.9260
**Logistic Regression (lr)**	0.5490	0.5012	0.5725	0.5309	0.5290	5.5390
**K-neighbors Classifier (knn)**	0.5003	0.4707	0.5099	0.4977	0.4770	0.7070
**Decision Tree Classifier (dt)**	0.4862	0.4512	0.4890	0.4872	0.4616	0.1590
**SVM – Linear kernel (svm)**	0.4473	0.4011	0.4671	0.4200	0.4227	0.2210
**Linear Discriminant Analysis**	0.4315	0.4094	0.4648	0.4165	0.4080	0.0470

(lda)						
Ridge Classifier (ridge)	0.3694	0.2679	0.3194	0.2809	0.3410	0.1300
Naïve Bayes (nb)	0.3143	0.3239	0.4796	0.2881	0.2969	0.0290
Quadratic discriminant Analysis (qda)	0.2284	0.2248	0.2299	0.1929	0.2178	0.0320
Ada Boost Classifier (ada)	0.2055	0.1928	0.2095	0.1530	0.1795	0.9500

Table S6. Scores of the different classifiers trained with the full dataset of 26 barcodes, sorted by higher accuracy first, using only the features extracted from the three main acquisitions channels (α, β, δ). TT = Total Time. Pycaret's automatic output.

Model	Accuracy	Balanced accuracy	Average precision	F1	MCC	TT (s)
Gradient Boosting Classifier (gbc)	0.4215	0.4235	0.4623	0.4149	0.4008	8.2880
Logistic Regression (lr)	0.4075	0.4102	0.5843	0.3876	0.3909	4.7630
Light Gradient Boosting Machine (lightgbm)	0.4063	0.4075	0.4321	0.4033	0.3840	0.9450
K-neighbors Classifier (knn)	0.3791	0.3801	0.4001	0.3779	0.3554	0.1370
MLP Classifier (mlp)	0.3562	0.3568	0.4428	0.3104	0.3397	0.7610
Random Forest Classifier (rf)	0.3561	0.3544	0.3586	0.3562	0.3304	0.4640
Extra Trees Classifier (et)	0.3504	0.3487	0.3526	0.3505	0.3244	0.3550
Decision Tree Classifier (dt)	0.3325	0.3310	0.3347	0.3326	0.3058	0.0230
Naïve Bayes (nb)	0.2985	0.2964	0.4345	0.2460	0.2848	0.0110
Linear Discriminant Analysis (lda)	0.2864	0.2826	0.3310	0.2646	0.2615	0.0120
Ridge Classifier (ridge)	0.2251	0.2218	0.1795	0.1432	0.2028	0.0080
SVM – radial kernel (rbfsvm)	0.1626	0.1633	0.1797	0.1454	0.1428	26.6900
SVM – Linear kernel (svm)	0.1436	0.1426	0.0784	0.0700	0.1221	0.2010
Ada Boost Classifier (ada)	0.1329	0.1325	0.0682	0.0602	0.1090	0.3240
Quadratic discriminant Analysis (qda)	0.0380	0.0385	0.0014	0.0028	0.0000	0.0070

Table S7. Scores of the different classifiers trained with the 10-class model, sorted by higher accuracy. TT = Total Time. Pycaret's automatic output.

Model	Accuracy	Balanced accuracy	Average precision	F1	MCC	TT (s)
MLP Classifier (mlp)	0.8550	0.8375	0.8572	0.8550	0.8365	4.3260
Light Gradient Boosting Machine (lightgbm)	0.8337	0.8150	0.8343	0.8332	0.8123	0.5140
Random Forest Classifier (rf)	0.8192	0.7996	0.8200	0.8181	0.7960	0.4240
Gradient Boosting Classifier (gbc)	0.8130	0.7940	0.8141	0.8120	0.7890	7.8200
Extra Trees Classifier (et)	0.8126	0.7933	0.8131	0.8111	0.7885	0.2360
SVM – radial kernel (rbfsvm)	0.7966	0.7725	0.8046	0.7950	0.7710	4.8650
Logistic Regression (lr)	0.7796	0.7572	0.7827	0.7782	0.7516	1.0870
K-neighbors Classifier (knn)	0.7595	0.7372	0.7592	0.7568	0.7288	0.2130
Decision Tree Classifier (dt)	0.7526	0.7328	0.7529	0.7523	0.7209	0.0530
SVM – Linear kernel (svm)	0.7477	0.7179	0.7549	0.7421	0.7165	0.0500
Linear Discriminant Analysis (lda)	0.6877	0.6730	0.7127	0.6892	0.6504	0.0240
Ridge Classifier (ridge)	0.6448	0.5696	0.6586	0.6224	0.6004	0.0160
Naïve Bayes (nb)	0.5599	0.5350	0.6595	0.5130	0.5232	0.0170
Ada Boost Classifier (ada)	0.4969	0.3915	0.3911	0.3879	0.4437	0.2840
Quadratic discriminant Analysis (qda)	0.4340	0.4394	0.2967	0.3397	0.3984	0.0180

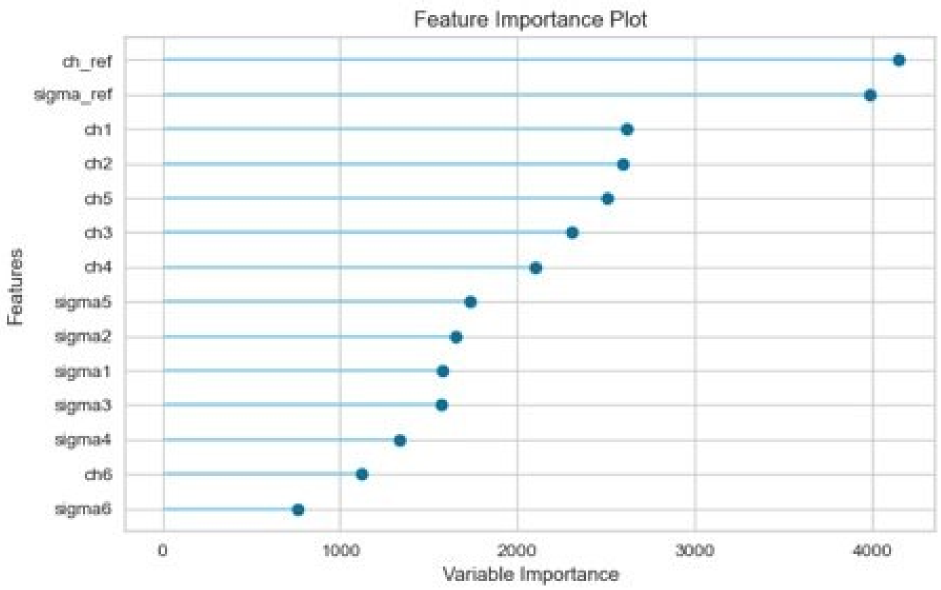

Fig. S9. Feature importance plot of the second-best performing model, a Light Gradient Boosting Machine. Pycaret's automatic output.

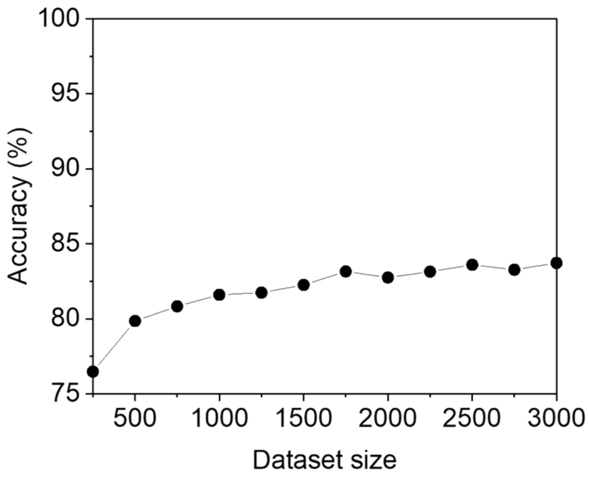

Fig. S10. Accuracy according to dataset size.

Table S8. Top-performance model, MLP classifier, hyperparameters before and after optimization. More information on these parameters can be found in Pycaret and scikit-learn's documentation. Highlighted in yellow, the hyperparameters that had changed.

Parameter	Value before optimization	Value after optimization	Explanation
**Activation function**	relu	relu	Activation function for the hidden layer.
**Alpha**	0.0001	0.001	Strength of the L2 regularization term. The L2 regularization term is divided by the sample size when added to the loss.
**Batch size**	Auto	Auto	Size of minibatches for stochastic optimizers.
**Beta 1**	0.9	0.9	Exponential decay rate for estimates of first moment vector in adam, should be in [0, 1). Only used when solver='adam'.
**Beta 2**	0.999	0.999	Exponential decay rate for estimates of second moment vector in adam, should be in [0, 1). Only used when solver='adam'.
**Early stopping**	False	False	Whether to use early stopping to terminate training when validation score is not improving. If set to true, it will automatically set aside 10% of training data as validation and terminate training when validation score is not improving by at least tol for n_iter_no_change consecutive epochs. The split is stratified, except in a multilabel setting. If early stopping is False, then the training stops when the training loss does not improve by more than tol for n_iter_no_change consecutive passes over the training set. Only effective when solver='sgd' or 'adam'.
**Epsilon**	1e-08	1e-08	Value for numerical stability in adam. Only used when solver='adam'.
**Hidden layer sizes**	(100,)	[50,50]	The ith element represents the number of neurons in the ith hidden layer.
**Learning rate**	Constant	adaptive	Learning rate schedule for weight updates.<br><br>'constant' is a constant learning rate given by 'learning_rate_init'.<br><br>'adaptive' keeps the learning rate constant to 'learning_rate_init' as long as training loss keeps decreasing. Each time two consecutive epochs fail to decrease training loss by at least tol, or fail to increase validation score by at least tol if 'early_stopping' is on, the current learning rate is divided by 5.
**Learning rate init**	0.001	0.001	The initial learning rate used. It controls the step-size in updating the weights.
**Max iterations**	500	500	Maximum number of iterations. The solver iterates until convergence (determined by 'tol') or this number of iterations. For stochastic solvers ('sgd', 'adam'), note that this determines the number of epochs (how many times each data point will be used), not the number of gradient steps.
**Number iterations (no change)**	10	10	Maximum number of epochs to not meet *tol* improvement. Only effective when solver='sgd' or 'adam'.
**Random state**	6971	6971	Determines random number generation for weights and bias initialization, train-test split if early stopping is used, and batch sampling when solver='sgd' or 'adam'. Pass

			an int for reproducible results across multiple function calls.
**Shuffle**	True	True	Whether to shuffle samples in each iteration.
**Solver**	adam	adam	The solver for weight optimization.
**Tol**	0.0001	0.0001	Tolerance for the optimization. When the loss or score is not improving by at least tol for n_iter_no_change consecutive iterations, unless learning_rate is set to ‘adaptive’, convergence is considered to be reached and training stops.
**Verbose**	False	False	Whether to print progress messages to stdout.
**Warm start**	False	False	When set to True, reuse the solution of the previous call to fit as initialization, otherwise, just erase the previous solution.

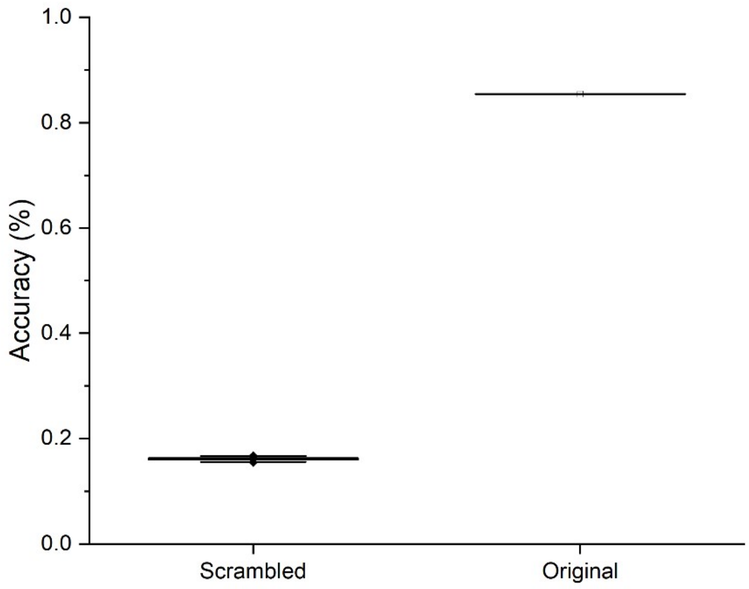

Fig. S11. y-scrambling analysis of the 10-class model. The analysis was performed shuffling the label column (Barcode ID) and re-training the model in 100 iterations.

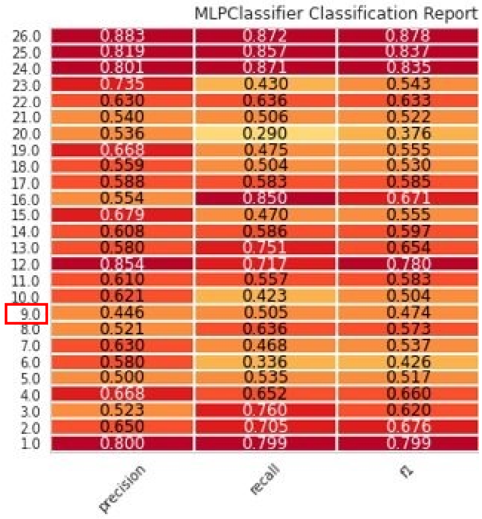

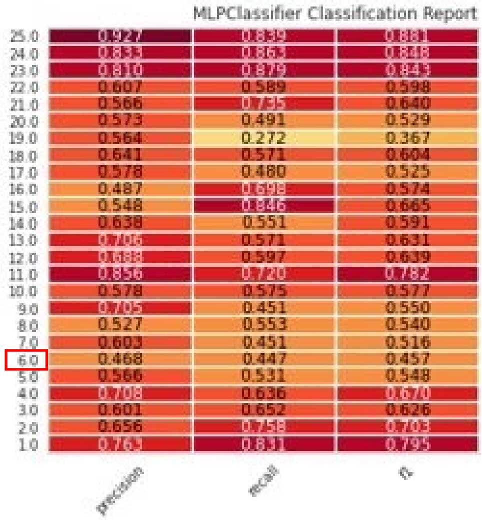

Class	Barcode
1	1
2	2
3	3
4	4
5	5
6	6
7	7
8	8
9	10
10	11
11	12
12	13
13	14
14	15
15	16
16	17
17	18
18	19
19	20
20	21
21	22
22	23
23	24
24	25
25	26

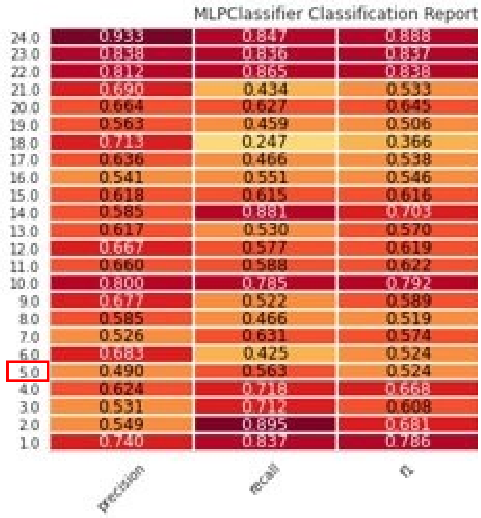

Class	Barcode
1	1
2	2
3	3
4	4
5	5
6	7
7	8
8	10
9	11
10	12
11	13
12	14
13	15
14	16
15	17
16	18
17	19
18	20
19	21
20	22
21	23
22	24
23	25
24	26

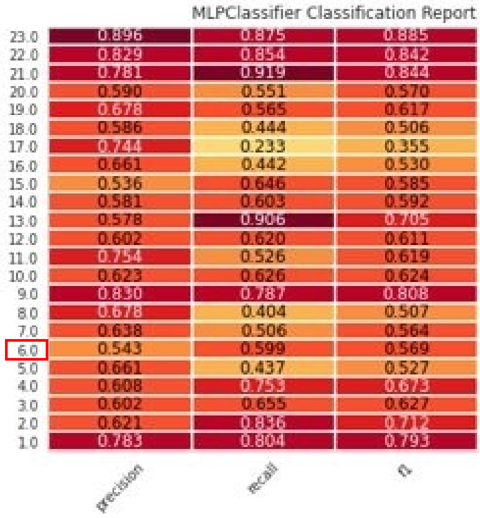

Class	Barcode
1	1
2	2
3	3
4	4
5	7
6	8
7	10
8	11
9	12
10	13
11	14
12	15
13	16
14	17
15	18
16	19
17	20
18	21
19	22
20	23
21	24
22	25
23	26

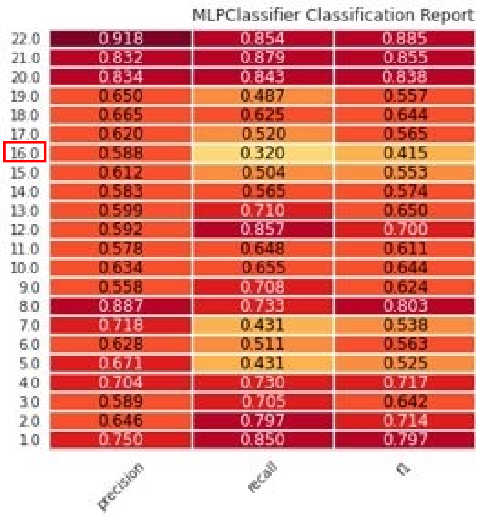

Class	Barcode
1	1
2	2
3	3
4	4
5	7
6	10
7	11
8	12
9	13
10	14
11	15
12	16
13	17
14	18
15	19
16	20
17	21
18	22
19	23
20	24
21	25
22	26

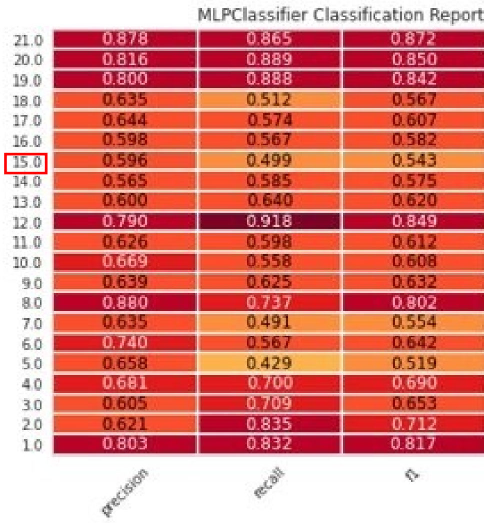

Class	Barcode
1	1
2	2
3	3
4	4
5	7
6	10
7	11
8	12
9	13
10	14
11	15
12	16
13	17
14	18
15	19
16	21
17	22
18	23
19	24
20	25
21	26

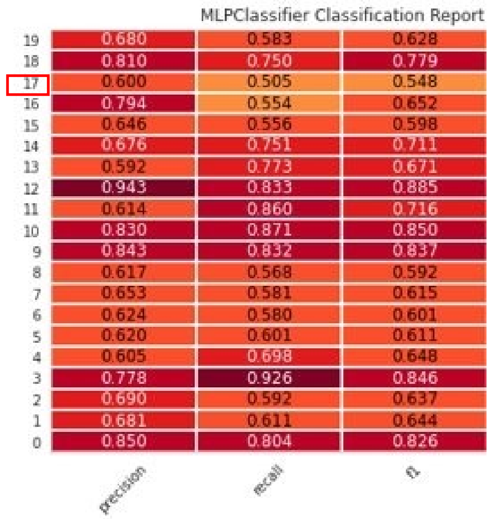

Class	Barcode
0	1
1	2
2	3
3	4
4	7
5	10
6	11
7	12
8	13
9	14

Class	Barcode
10	15
11	16
12	17
13	18
14	21
15	22
16	23
17	24
18	25
19	26

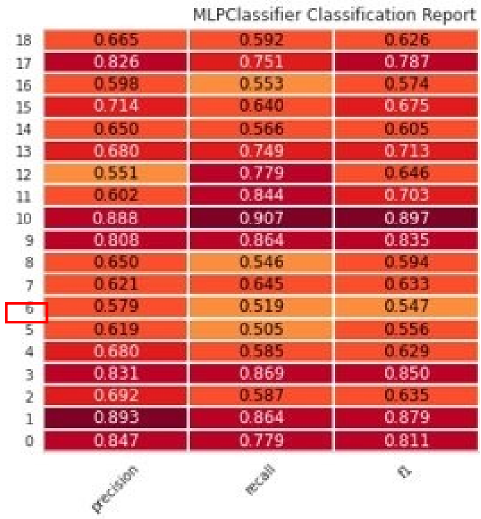

Class	Barcode
0	1
1	2
2	3
3	4
4	7
5	10
6	11
7	12
8	13
9	14

Class	Barcode
10	15
11	16
12	17
13	18
14	21
15	22
16	23
17	25
18	26

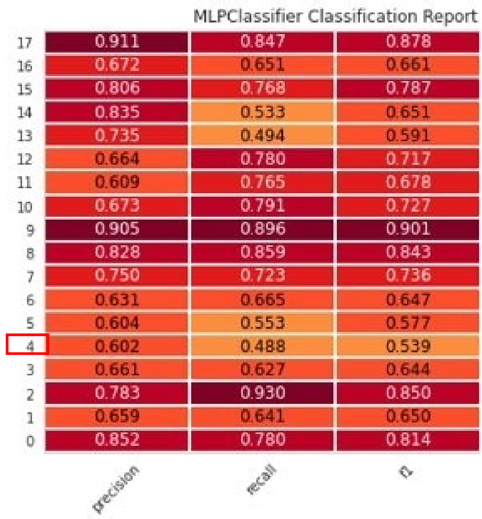

Class	Barcode
0	1
1	2
2	3
3	4
4	7
5	10
6	12
7	13
8	14

Class	Barcode
9	15
10	16
11	17
12	18
13	21
14	22
15	23
16	25
17	26

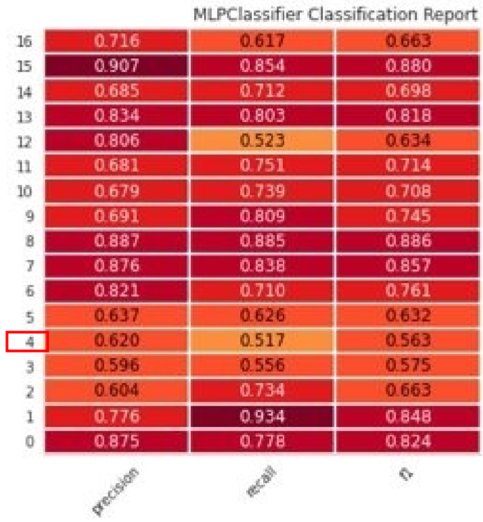

Class	Barcode
0	1
1	2
2	3
3	4
4	10
5	12
6	13
7	14
8	15

Class	Barcode
9	16
10	17
11	18
12	21
13	22
14	23
15	25
16	26

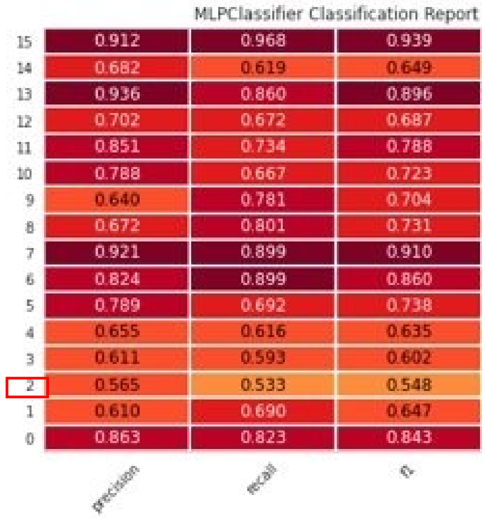

Class	Barcode
0	1
1	2
2	3
3	4
4	12
5	13
6	14
7	15

Class	Barcode
8	16
9	17
10	18
11	21
12	22
13	23
14	25
15	26

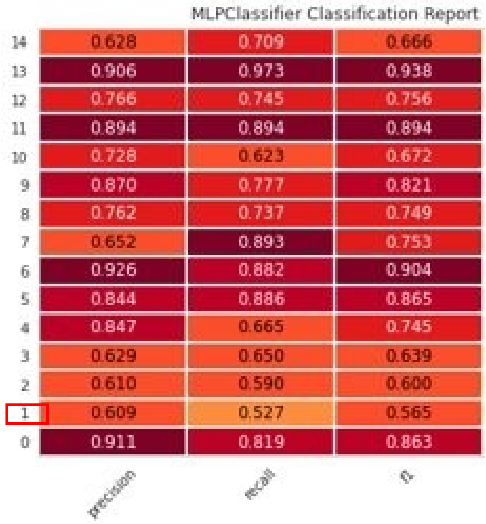

Class	Barcode
0	1
1	2
2	4
3	12
4	13
5	14
6	15
7	16

Class	Barcode
8	17
9	18
10	21
11	22
12	23
13	25
14	26

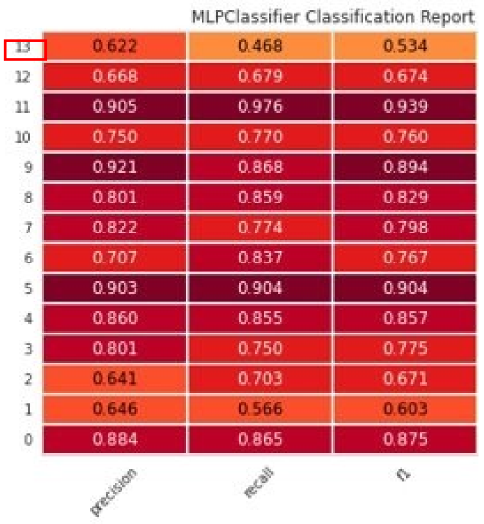

Class	Barcode
0	1
1	4
2	12
3	13
4	14
5	15
6	16

Class	Barcode
7	17
8	18
9	21
10	22
11	23
12	25
13	26

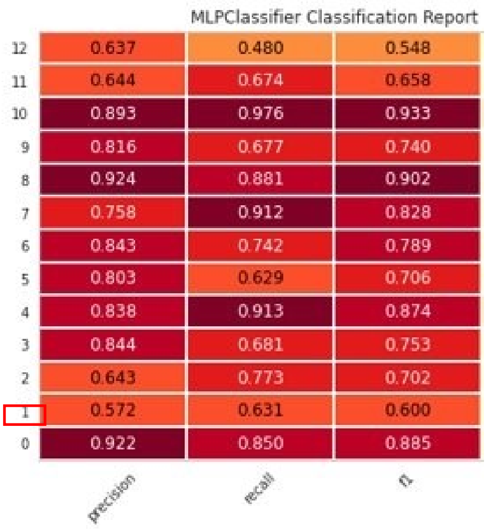

Class	Barcode
0	1
1	4
2	12
3	13
4	14
5	15
6	16

Class	Barcode
7	17
8	18
9	21
10	22
11	23
12	25

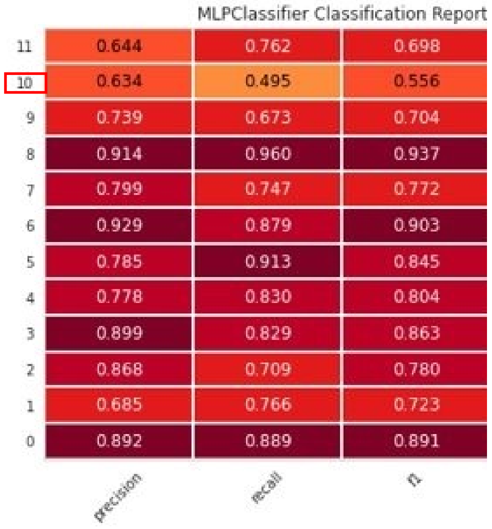

Class	Barcode
0	1
1	12
2	13
3	14
4	15
5	16

Class	Barcode
6	17
7	18
8	21
9	22
10	23
11	25

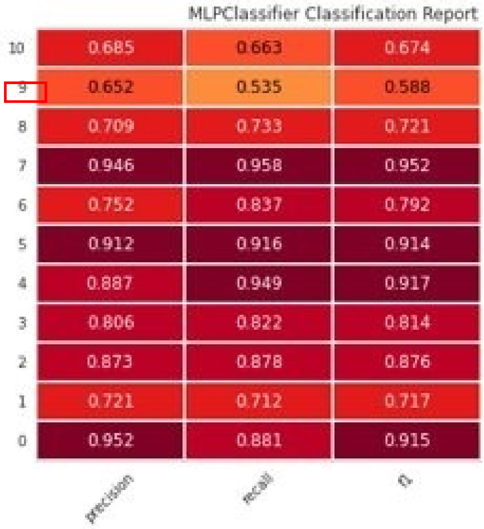

Class	Barcode
0	1
1	12
2	13
3	14
4	15
5	16

Class	Barcode
6	17
7	18
8	21
9	22
10	25

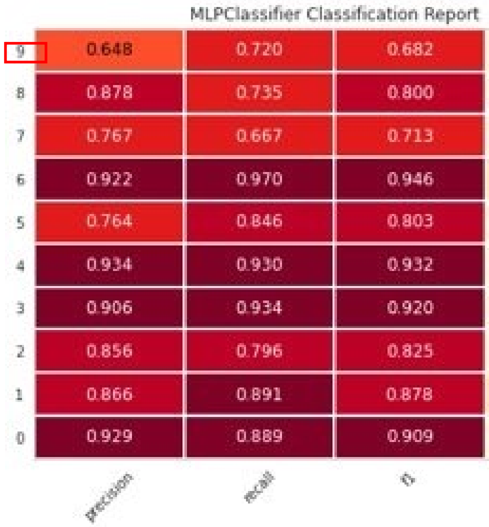

Class	Barcode
0	1
1	12
2	13
3	14
4	15

Class	Barcode
5	16
6	17
7	18
8	21
9	25

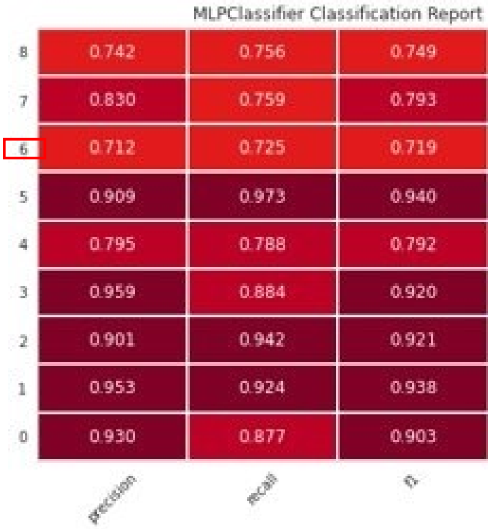

Class	Barcode
0	1
1	12
2	13
3	14
4	15

Class	Barcode
5	16
6	17
7	18
8	21

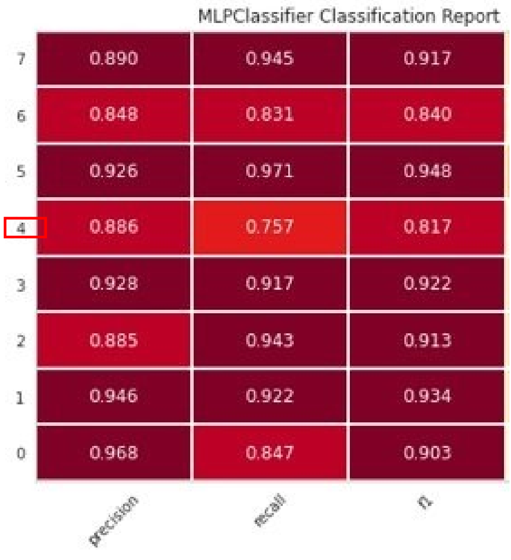

Class	Barcode
0	1
1	12
2	13
3	14
4	15
5	16
6	18
7	21

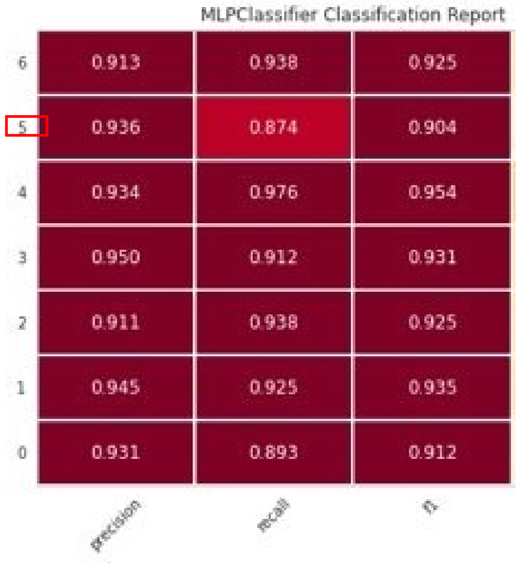

Class	Barcode
0	1
1	12
2	13
3	14
4	16
5	18
6	21

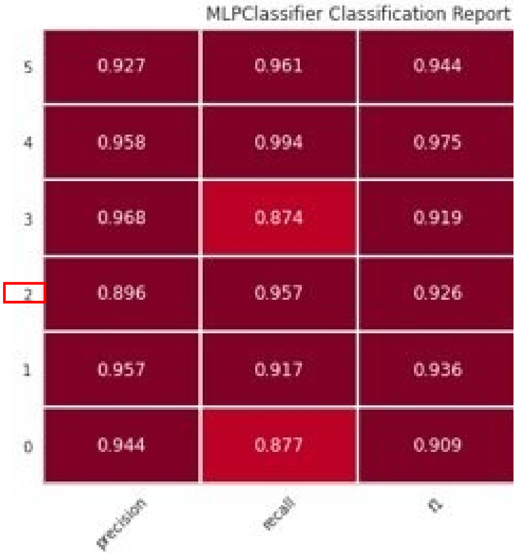

Class	Barcode
0	1
1	12
2	13
3	14
4	16
5	21

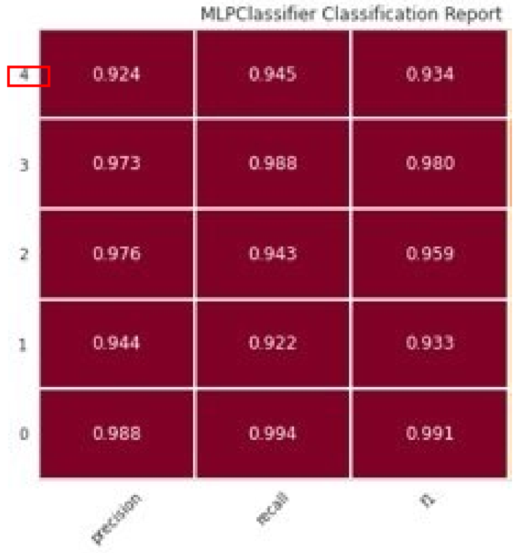

Class	Barcode
0	1
1	12
2	14
3	16
4	21

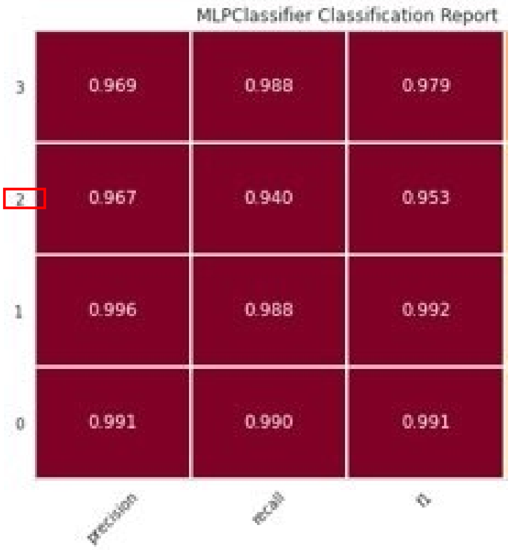

Class	Barcode
0	1
1	12
2	14
3	16

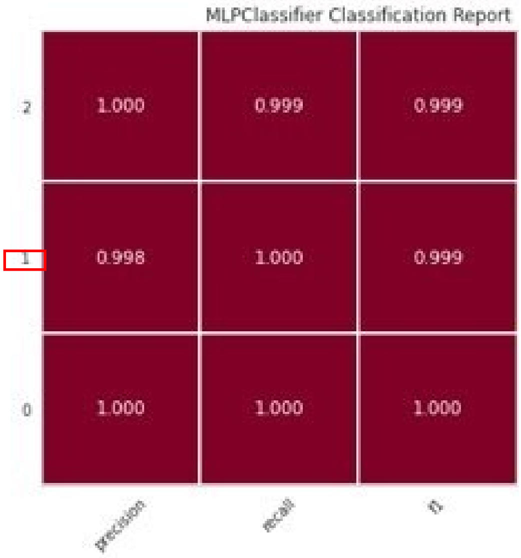

Class	Barcode
0	1
1	12
2	16

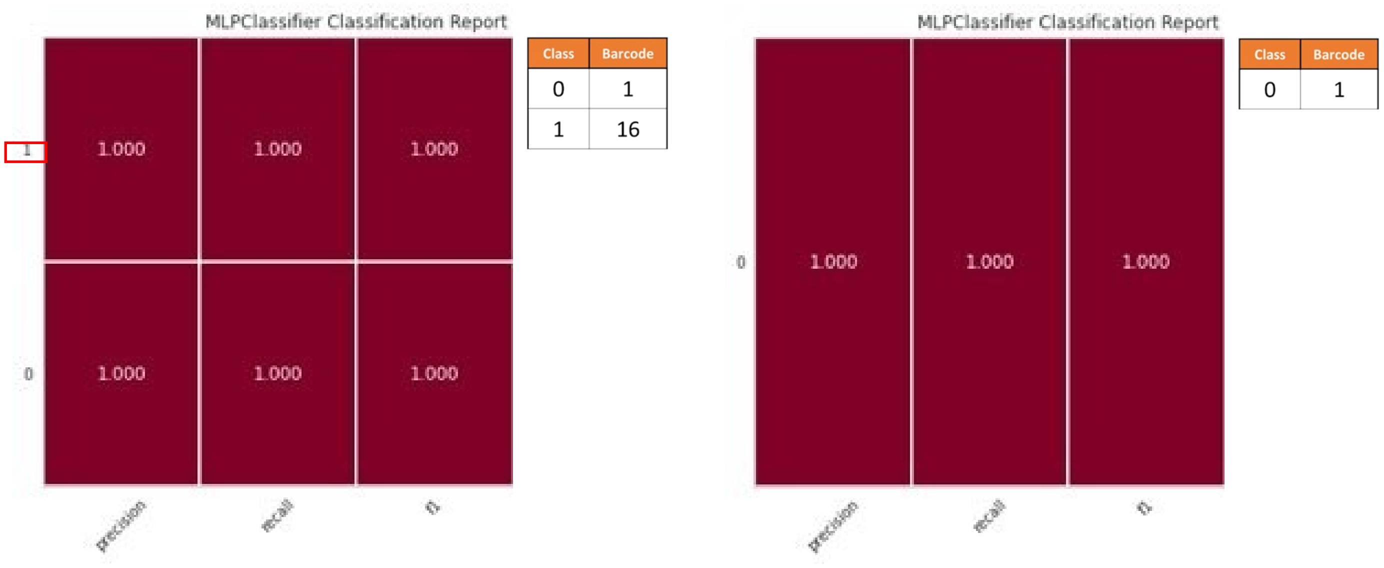

Fig. S12. Metrics per class for each of the 26 models trained to study the trade-off between accuracy and number of classes. Next to them, the correspondence between the class and the barcode they represent. Recall is synonim to sensitivity.

Table S9. Model stability analysis. Performing the random splits (80% training - 20% testing) over 5 iterations on the same dataset generates 5 10-classes models. The average precision and sensitivity on predictions made using the training and testing sets are given below. This way the models stability and any potental overfitting are assessed.

Barcode ID		Training set		Testing set	
		**Mean**	**SD**	**Mean**	**SD**
**1**	Precision	0.94	0.01	0.92	0.01
	Sensitivity	0.93	0.02	0.93	0.02
**12**	Precision	0.88	0.02	0.83	0.02
	Sensitivity	0.89	0.02	0.87	0.01
**13**	Precision	0.89	0.02	0.90	0.02
	Sensitivity	0.93	0.02	0.91	0.01
**14**	Precision	0.96	0.01	0.97	0.01
	Sensitivity	0.91	0.02	0.88	0.02
**15**	Precision	0.82	0.04	0.82	0.03
	Sensitivity	0.84	0.02	0.85	0.02
**16**	Precision	0.90	0.01	0.89	0.01
	Sensitivity	0.94	0.01	0.94	0.01
**17**	Precision	0.72	0.02	0.69	0.02
	Sensitivity	0.81	0.03	0.75	0.03
**18**	Precision	0.83	0.02	0.83	0.02
	Sensitivity	0.80	0.04	0.79	0.02
**21**	Precision	0.74	0.02	0.73	0.02
	Sensitivity	0.67	0.05	0.68	0.05
**25**	Precision	0.91	0.03	0.86	0.03
	Sensitivity	0.85	0.03	0.81	0.03
**Global**	Balanced accuracy	0.86	0.03	0.84	0.00
